# The Impact of Pleistocene Glacial Cycles on the Evolutionary Diversification of the Arctic‐Alpine *Silene acaulis* Species Complex

**DOI:** 10.1111/mec.70254

**Published:** 2026-01-23

**Authors:** Oliver Reutimann, Gwyneth Halstead‐Nussloch, Andreas Tribsch, Pablo Tejero Ibarra, Niklaus Zemp, Alex Widmer, Martin C. Fischer

**Affiliations:** ^1^ Institute of Integrative Biology ETH Zurich Zurich Switzerland; ^2^ Department of Environment and Biodiversity University of Salzburg Salzburg Austria; ^3^ Herbarium JACA, Pyrenean Institute of Ecology‐CSIC Jaca Spain; ^4^ Genetic Diversity Centre (GDC), ETH Zurich Zurich Switzerland

**Keywords:** admixture, colonisation, ddRAD, introgression, lineage diversification, molecular dating, reference genome

## Abstract

Arctic‐alpine species are highly sensitive to long‐term temperature changes and associated glacial cycles due to their occurrence in cold environments to which they are adapted and spatially restricted. Unravelling their evolutionary responses to past climatic fluctuations can provide new insights into their diversification. In this study, we investigated the evolutionary history of the *Silene acaulis* species complex and how it was shaped by past glacial cycles. We assembled the first high‐quality reference genome for *S. acaulis* and analysed reduced representation sequencing data from 955 individuals spanning 132 populations across the Holarctic distribution range of these arctic‐alpine cushion plants. We identified five evolutionary lineages and assessed their phylogeographical structure in relation to current subspecies classifications, refugia, and historic migration patterns. Phylogenetic dating revealed that lineage divergence largely coincided with repeated phases of glacial cooling over the last two million years and was driven by isolation in glacial refugia. Secondary contact in glacial refugia or during interglacial expansions promoted hybridization and further shaped the distribution of genetic diversity across the species complex. Adaptive divergence amongst sympatric genetic groups in the European Alps highlights the contribution of niche specialisation to intraspecific divergence, with evidence for ecotype differentiation in response to a combination of edaphic and climatic factors. The *S. acaulis* species complex has an intricate evolutionary history, shaped by glacial cycles in the Late Pleistocene that have driven lineage diversification, secondary contact and ecotype formation. Our study underscores the significance of glacial cycles in shaping genetic diversity in arctic‐alpine plant species and improves our understanding of how arctic‐alpine species have responded to past climate fluctuations.

## 1 Introduction

Throughout the Pleistocene, periods of glacial cooling were followed by interglacial warming in response to recurrent climatic oscillations driven by Milankovitch cycles (Bennett 1990). These climatic shifts have profoundly impacted biodiversity by altering ecosystems, the composition of communities, and genetic variation within and amongst species (Comes and Kadereit 1998; Hewitt 2000, 1996; Rahbek et al. 2019). Arctic‐alpine plant species were particularly affected by these climatic cycles and experienced dramatic range shifts, as they are adapted to narrow ecological niches in environments defined by cold temperatures and short growing seasons (Abbott 2008; Billings and Mooney 1968). During glacial expansions, arctic‐alpine plant species were directly affected by the increasing ice cover, and their survival relied on migration towards more suitable habitats and the presence of refugia both at the periphery of and within glaciated areas (Holderegger and Thiel‐Egenter 2009; Schönswetter et al. 2005). Isolation of populations in refugia can lead to genetic drift, in particular when refugial populations are small and isolated for long periods of time, and underlie genetic divergence amongst refugial populations (Kadereit 2023; Rota et al. 2024; Stewart et al. 2010). However, natural selection driven by specific environmental conditions in refugia can also mediate adaptation to local environmental conditions. This facilitates the formation of ecotypes, particularly in highly heterogeneous landscapes such as mountain ranges (Halbritter et al. 2018; Kadereit 2023; Maier et al. 2019).

During warming periods, retreating glaciers allowed populations to expand their ranges by colonising areas that had previously been covered by ice. In the process, they adapted to the new climatic conditions (Luqman et al. 2023). These repeated recolonizations have likely facilitated secondary contact and genetic admixture between previously isolated populations (Hewitt 2000; Petit et al. 2003). In Europe, the Alps are a hotspot for secondary contact zones between plant lineages, as alpine species recolonized the Alpine Arc after the last glacial maximum (LGM) from peripheral refugia surrounding the Alps or from nunatak refugia on ice‐free mountain peaks (Holderegger and Thiel‐Egenter 2009; Schönswetter et al. 2003; Thiel‐Egenter et al. 2009). Similar cases of secondary contact zones are also known from Arctic areas such as Svalbard. This island was recolonized by several plant species from multiple refugia following the LGM (Alsos et al. 2007; Brožová et al. 2023). Such genetic exchange amongst populations following recolonization may partly homogenise accumulated genetic differences if reproductive isolation is insufficient to prevent gene flow (Kadereit 2023; Parisod 2022). Moreover, there is potential to exchange adaptive alleles through introgression, facilitating adaptation to novel environments. The fusion of evolutionary lineages following secondary contact may even give rise to hybrid lineages that have the potential to occupy new environmental niches (Mallet 2007).

The retreat of glaciers during warmer climatic periods subsequently altered the composition of plant communities, leading to the gradual replacement of cold‐adapted species by thermophilic ones (Abbott 2008; Alexander et al. 2015). In response, arctic‐alpine species dispersed to newly deglaciated land at high latitudes or altitudes to which they are adapted (Watts et al. 2022). Such range shifts pose a growing challenge for cold‐adapted species, as observed under current climate change (Iseli et al. 2023). As cold‐adapted plant communities migrate upwards in latitude or altitude, the availability of suitable habitat for these species becomes increasingly limited, threatening their long‐term survival (Birks 2008; Watts et al. 2022). If warming continues at predicted rates, many arctic‐alpine plant species are at risk of extinction due to the predicted loss of their habitats (Birks 2008; Niskanen et al. 2019; Wessely et al. 2022). To cope with these challenges, arctic and alpine species have to rely on the heterogeneity of arctic and alpine environments, and on their adaptive capacity for responding to novel climatic conditions. While current climate change poses significant challenges, it is important to recognise that cold‐adapted floras have historically persisted through repeated climatic cycles. The repeated separation of populations by growing ice sheets during cooling periods and the formation of novel contact zones during warmer periods has contributed to their taxonomic complexity, increased rates of divergence, dispersal, hybridization, and speciation observed in cold‐adapted floras (Abbott and Brochmann 2003; Brochmann and Brysting 2008; Karl et al. 2012).

The emblematic arctic‐alpine plant species *Silene acaulis* (L.) Jacq. is a perennial, cushion‐forming plant in the Caryophyllaceae family. Its arctic‐alpine distribution includes regions of Europe, North America, and Asia; however, there is a large distribution gap in Siberia (Jones and Richards 1962). *Silene acaulis* shows high levels of intraspecific genetic diversity and forms a species complex with four subspecies: *S. acaulis* subsp. *acaulis* (L.) Jacq. [= subsp. *longiscapa* (Vierh.)], subsp. *subacaulescens* (N. F. Williams) Hultén, subsp. *exscapa* (All.) Braun Blanq. [= subsp. *bryoides*; (Jordan) Nyman], and subsp. *cenisia* (Vierh.).

The taxonomic delimitation of intraspecific taxa within the *S. acaulis* species complex has yet to be settled. *Silene acaulis* subsp. *subacaulescens* is primarily found in Beringia and North America, whereas all other subspecies are distributed across Europe, where they partly occur in sympatry (Lauber et al. 2024). The taxonomic assignment of individual plants to subspecies based on morphology alone can be challenging, as subspecies display broad and sometimes overlapping morphological variation. However, there is ample evidence, also from field botanists, indicating ecological niche differentiation (Chardon et al. 2020) and distinct edaphic preferences, in particular in the western Alps, where subsp. *exscapa* grows on siliceous bedrock and subsp. *acaulis* on calcareous bedrock (Aeschimann et al. 2004; Maurice et al. 1998). These two taxa are even considered different species in Switzerland: *S. acaulis* (L.) Jacq., and *S. exscapa* All. (Lauber et al. 2024). In addition to morphology, extensive variation in sexual systems has been observed in the *S. acaulis* species complex. The widely distributed subsp. *acaulis* has been reported to be gynodioecious or trioecious (a sexual system in which males, females, and hermaphrodites coexist) in northernmost Sweden (Alatalo 1997; Alatalo and Molau 1995, 2001), Svalbard (Svoen et al. 2019), Greenland (Philipp et al. 2009), the Swiss Alps and the Pyrenees (Canelles et al. 2018). Subspecies *subacaulescens* has been reported to be gynodioecious in the Rocky Mountains of western North America (Delph and Carroll 2001; Delph 2004; Shykoff 1988; Städler and Delph 2002), as well as on Baffin Island (Hermanutz and Innes 1994), British Columbia in Canada (Reid et al. 2014), and Alaska (Keller and Schwaegerle 2006; Klaas and Olson 2006). Additionally, the two sympatric subsp. *exscapa* and subsp. *cenisia* are reported to be dioecious and trioecious, respectively (Desfeux et al. 1996; Maurice et al. 1998).

Cold‐adapted cushion plants, such as *S. acaulis* , are an integral part of arctic‐alpine ecosystems. They endure not only harsh conditions but also serve as facilitators, nurse plants, and foundation species for other plants, arthropods, and soil microorganisms (Alatalo and Little 2014). *S. acaulis* cushions mitigate stress conditions at the microclimatic level for other plants and invertebrates and increase arthropod diversity in alpine ecosystems (Molenda et al. 2012). Thus, *S. acaulis* is a key species of arctic‐alpine ecosystems. At the same time, it has been shown that *S. acaulis* may be sensitive to climate change in the Alps (Rai et al. 2025). Under the RCP2.6 climate change scenario, it is predicted to lose 50% of its range size, only 10% of which can be compensated by shifting upslope (Wessely et al. 2022). Additional pressure comes from the anther‐smut pathogen *Microbotryum silenes‐acaulis*, which causes anther‐smut disease across the entire range of *Silene acaulis* and shows higher prevalence at northern latitudes (Bueker et al. 2016). Because of its climate sensitivity, arctic‐alpine distribution, host‐pathogen dynamics, edaphic adaptation, and sexual system variation, the *S. acaulis* species complex is an ideal system to study a wide range of evolutionary and ecological questions. Addressing such questions requires detailed knowledge about the phylogeographic history of this species complex and the delineation of evolutionary lineages and ecotypes.

An earlier study, based on AFLP markers and plastid DNA sequences, focused on *S. acaulis* subsp. *acaulis* and subsp. *subacaulescens* and revealed first insights into the complex phylogeographic history for the two subspecies, with their range‐wide distribution shaped by vicariance, long‐distance dispersal, extinction, and recolonization from multiple refugia (Gussarova et al. 2015). The study further provided some evidence for two genetic groups in North America and two genetic groups in Europe, likely associated with different refugia. The crown age of subsp. *acaulis* and subsp. *subacaulescens* was dated to around 0.93 million years ago (Gussarova et al. 2015), which coincides with the Mid‐Pleistocene Transition and is consistent with an independent phylogenetic analysis of the genus *Silene* by Petri et al. (2013). However, these earlier analyses did not fully capture the intraspecific genetic diversity of the *S. acaulis* species complex, as the European Alps, a recognised biodiversity hotspot (Chauvier‐Mendes et al. 2024; Sabatini et al. 2022) and refugium for arctic‐alpine species during warming periods (Stewart et al. 2010), were not adequately sampled.

The European Alps lie at the centre of diversity within the *S. acaulis* complex. Two subspecies occur exclusively in the European Alps and differ in their sexual systems and edaphic preferences: subsp. *exscapa* is dioecious and grows on siliceous bedrock, whereas subsp. *cenisia* is trioecious and prefers calcareous bedrock (Aeschimann et al. 2004; Maurice et al. 1998). Consequently, our understanding of the evolutionary relationships within the *S. acaulis* species complex remains incomplete as the diversity of the European Alps has previously been inadequately covered. Examining the role of past climatic shifts in shaping the evolutionary history of the *S. acaulis* species complex, along with adaptive and sexual system divergence, can provide valuable insights into the mechanisms driving lineage diversification, early speciation, and species' responses to changing climatic conditions.

In this study, we examined the evolutionary history of the *S. acaulis* species complex by *de novo* assembling its first reference genome and analysing double‐digest restriction‐site associated DNA (ddRAD) sequencing data from 955 individuals across 132 populations. Our sampling covered a significant portion of the species' Holarctic distribution, with a particular focus on the European Alps. To gain a detailed understanding of the evolutionary history of the species complex, we first identified evolutionary lineages and compared them to the current concept of described subspecies. We further assessed the genetic structure within each lineage to identify potential colonisation routes. Secondly, we inferred the evolutionary divergence times between lineages and examined how they aligned with Pleistocene glacial cycles. Thirdly, we assessed the impact of secondary contact, hybridization, and introgression on the evolution of different lineages and taxa. Finally, we explored how adaptive differentiation contributed to the evolutionary diversification of partly sympatric genetic groups in the Alpine Arc.

## 2 Materials and Methods

### 2.1 De Novo *Assembly of the Silene acaulis Reference Genome*

We *de novo* assembled a haplotype phased reference genome (eth_SilAcau_F_GE_1) from a single female *S. acaulis* individual from Switzerland (S_ac_CH_Ge_11; 46.397° N, 7.606° E, see Table S1). High molecular weight DNA was extracted from flash‐frozen leaf tissue followed by library preparation for PacBio HiFi sequencing on the Revio platform at Novogene (Novogene Company Limited, Cambridge, UK), as well as Omni‐C library generation and paired‐end sequencing (Dovetail Genomics/Cantata Bio, CA, USA). HiFi and Omni‐C reads were used to produce haplotype phased assemblies with hifiasm (Cheng et al. 2022) using purging parameter ‘‐l 3’ and Omni‐C reads further applied for scaffolding using YaHS (Zhou et al. 2023) and manual curation using PretextView (https://github.com/sanger‐tol/PretextView). For the annotation, we used the BRAKER3 pipeline (Gabriel et al. 2024) to predict protein coding gene models. More detailed information can be found in the Supporting Information.

### 2.2 Sampling

**FIGURE 1:**
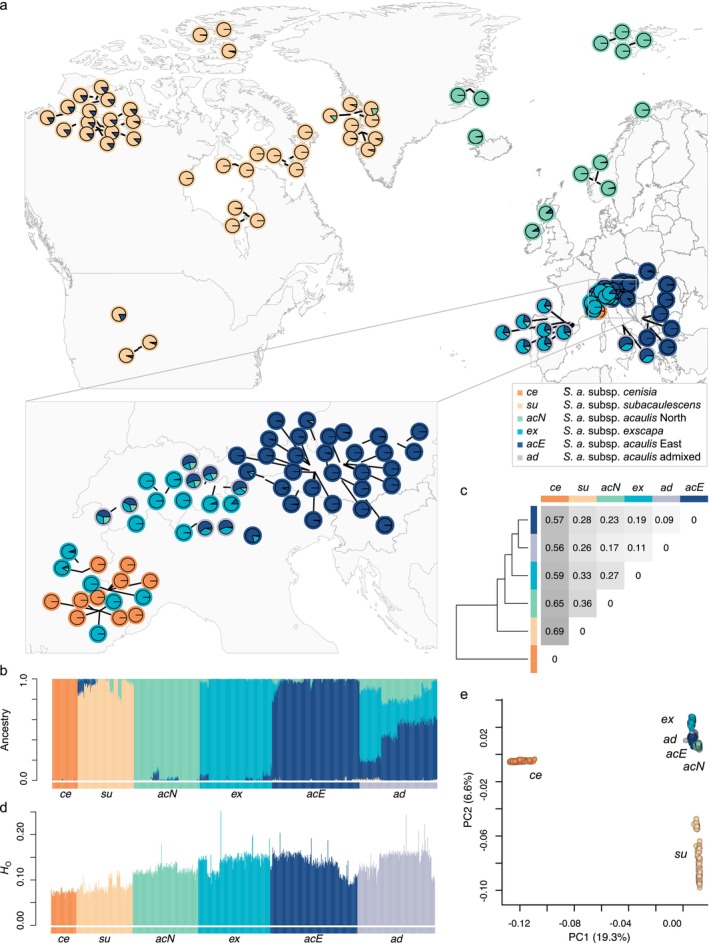
Evolutionary lineages and admixture within the *Silene acaulis* species complex inferred using 28,666 SNPs and 955 individuals from 132 populations. (a) Map showing the geographic distribution of the sampled populations, with each population represented by a pie chart indicating the ancestry assignments for *K* = 5 genetic clusters (i.e., evolutionary lineages). (b) Individual ancestry proportions for each genetic group (*K* = 5). Individuals are arranged from east to west within each group. Admixed samples entail individuals with less than 75% ancestry assignment to any of the evolutionary lineages. (c) Pairwise *F*_ST_ values between genetic groups. (d) Individual observed heterozygosity (*H*_O_) in each genetic group. (e) Principal component analysis (PCA) based on genetic data of 955 individuals. The variance explained by each principal component is indicated in parentheses.

This study included 955 individuals with high quality genetic data from the *S. acaulis* species complex, representing 132 populations across most of its distribution range, with a particular focus on Europe (see Figure 1a, Figure S1 and Table S1 for details). Initially 1032 individuals were genotyped, but 77 have been excluded because of low coverage or bad quality. Samples were assigned based on their morphology and geographical origin to one of the four subspecies of *S. acaulis* : subsp. *acaulis*, subsp. *subacaulescens*, subsp. *exscapa*, or subsp. *cenisia*.

### 2.3 ddRAD Genotyping

Individual DNA was extracted from dried leaf tissue following an in–house protocol using the LGC Genomics Sbeadex plant extraction kit on a Kingfisher instrument (Thermo Fischer Scientific, MA, USA). DNA quantification was performed using the QuantiFluor ONE dsDNA dye (Promega, Madison, WI, USA) on a Spark 10 M plate reader (Tecan, Männedorf, Switzerland). Library preparation followed a customised ddRAD protocol (Tschan et al. 2026). After multiplexing, 2 × 150 bp paired‐end sequencing was performed on an Illumina NovaSeq 6000 by Novogene (Novogene Company Limited, Cambridge, United Kingdom). The 9 negative controls did not yield any raw reads. Raw reads from the remaining samples were demultiplexed using the default settings of ‘process_radtags’ from Stacks v2.41 (Catchen et al. 2013) and mapped onto the newly assembled hap1 *S. acaulis* reference genome (eth_SilAcau_F_GE_1.1) using the default settings of BWA‐mem2 v2.2.1 (Li and Durbin 2009). Low‐quality hits (mapping quality < 20) and non‐primary hits were removed using samtools v1.20 (Danecek et al. 2021).

We used Freebayes v1.3.6 (Garrison and Marth 2012) to call variant sites using the following parameters: ‐p 2 ‐F 0.05 ‐C 1 ‐E −1 ‐‐use‐best‐n‐alleles 4 ‐‐haplotype‐length −1. Initially, we validated the sequencing data quality by performing variant calling and filtering (using similar filters as described below) on 27 technical replicates across two chromosomes and inferred genotyping error rates using Tiger (Bresadola et al. 2020). After successful validation (global genotyping error rate: 0.35%), variants were called on all 12 chromosomes using the entire dataset. We differentiated two datasets for subsequent analysis: a ‘complete SNP set’ including *S. acaulis* subsp. *cenisia*, and a ‘core SNP set’ that excludes *S. acaulis* subsp. *cenisia*. For the complete SNP set, we set the missingness per SNP to 5% and for the core SNP, we set it to 10%. This more stringent approach for the first SNP set was necessary to address the relatively high SNP missingness in the divergent *S. acaulis* subsp. *cenisia* individuals by including only SNPs in the dataset that were also found in *S. acaulis* subsp. *cenisia*. For the filtering itself, we applied a minor allele count threshold of 3, minimum mean depth of 8, removed complex multi‐nucleotide polymorphisms, and retained only biallelic SNPs. Individuals with too high missing rates were excluded in both data sets, that is, exceeding 35% in the complete SNP set and 50% in the core SNP set, resulting in the 955 individuals analysed in this study. Detailed information and additional filtering steps, inspired by the dDocent filtering pipeline (Puritz et al. 2014), are outlined in Table S2.

### 2.4 Genetic Structure and Evolutionary Lineages

To investigate the genetic structure and the evolutionary history of the *S. acaulis* species complex, we combined a set of population genetic and phylogeographic analyses. First, we identified genetic clusters using the complete SNP set and ADMIXTURE (Alexander et al. 2009) with a 10‐fold cross‐validation for *K* = 1–10 genetic clusters and ten repetitions for each *K*. We summarised the results using CLUMPAK (Kopelman et al. 2015). The number of *K* genetic clusters (i.e., evolutionary lineages) used for subsequent individual assignment was determined by visual inspection of log likelihood values and cross‐validation errors. Individuals with ancestry proportion higher than 0.75 were assigned to the corresponding evolutionary lineage and the individuals not fitting this criterion were classified as the ‘admixed group’. To quantify the genetic differentiation amongst the identified evolutionary lineages, we calculated the genetic fixation index *F*_ST_ (Weir and Cockerham 1984) using the *snpgdsFST* function from the SNPRelate package (Zheng et al. 2012) in R version 4.1.2 (R Core Team 2021). Individual observed heterozygosity (*H*_O_) was calculated for each sample as the proportion of heterozygous sites relative to the total number of non‐missing sites based on the genotype table, using R. We assessed significant differences in *H*_O_ amongst evolutionary lineages using analysis of variance (ANOVA) via the *aov* function and performed post hoc comparisons of group means with Tukey's HSD test in R using the *TukeyHSD* function. To visualise genetic variation amongst individuals and to confirm evolutionary lineages and admixed groups identified in the ADMIXTURE analysis, we conducted a principal component analysis (PCA) using the *snpgdsPCA* from SNPRelate (Zheng et al. 2012). To investigate further substructure within the *S. acaulis* species complex, we performed PCAs separately for each evolutionary lineage and the admixed group using the core SNP set. Finally, we produced minimum spanning trees based on pairwise population *F*_ST_‐values via the *mst* function of the ape R‐package (Paradis and Schliep 2019) and visualised these trees on a map.

### 2.5 Phylogenetic Reconstruction, Divergence Times and Secondary Contact

To explore phylogenetic relationships, divergence times and detect signatures of historical introgression, we selected a subset of 48 individuals representing a balanced design based on the five identified evolutionary lineages and the admixed group with 8 individuals each. We created two new SNP sets, which were filtered in the same way as the complete SNP set. The first SNP set consisted of all 48 selected individuals, while the second SNP set contained 40 individuals, excluding admixed individuals, as they can violate assumptions made for the inference of bifurcating phylogenetic trees. Additionally, we produced linkage disequilibrium (LD) pruned SNPs with no missing data using the *snpgdsLDpruning* function of the SNPRelate package (Zheng et al. 2012) in R with the ‘corr’ method, ld.threshold = 0.2 and missing.rate = 0. We then constructed unrooted phylogenetic networks using the Neighbour Net algorithm in SplitsTree (Huson and Bryant 2024) for each of the two LD‐pruned SNP sets to investigate evolutionary relationships.

To infer divergence times and phylogenetic relationships while incorporating incomplete lineage sorting, we performed coalescent‐based Bayesian tree inference using SNAPPER (Stoltz et al. 2021), an add‐on package to BEAST2.5 (Bouckaert et al. 2019) based on the LD‐pruned SNPs of the 40 populations assigned to evolutionary lineages, excluding admixed populations. SNAPPER estimates phylogenies directly from unlinked SNPs by modelling allele‐frequency changes along branches under a Wright–Fisher diffusion, effectively integrating over all possible gene trees (Bryant et al. 2012; Stoltz et al. 2021). We used a strict‐clock model that can be time calibrated using generally applicable priors (Stange et al. 2018). We then used the ‘snapp_prep.rb’ script (Stange et al. 2018) to produce an xml input file for BEAST, specifying an age constraint on the crown of all individuals, except those belonging to *S. acaulis* subsp. *cenisia*, modelled as a lognormal distribution with a mean of 0.935 million years ago (mya) and a standard deviation of 0.06. This age constraint corresponds to the crown age of *S. acaulis* , estimated by Gussarova et al. (2015) as having a mean of 0.935 mya and a 95% highest posterior density (HPD) interval of 0.826–1.104 mya. A phylogenetic tree of the genus *Silene* further provided independent support for the divergence of *S. acaulis* populations around the same time, estimating the divergence between a Swedish (botanical garden) and a Pyrenean sample of *S. acaulis* at 0.927 mya (95% HPD 0.146–1.852; Petri et al. 2013). Calibration points were obtained from previously published studies that themselves calibrated phylogenies using established sources. While this approach inherently carries some uncertainty due to propagation of assumptions from the original calibrations, it represents the most suitable temporal framework available for our dataset in the absence of direct fossil calibrations. Notably, two independent studies arrived at highly similar estimates, supporting the reliability of these calibration points for our analysis. We ran the model for 5,000,000 MCMC iterations with a burn‐in of 10%, performing two replicate runs. Convergence of the MCMC was assessed with the software Tracer (Rambaut et al. 2018), with all effective sample size (ESS) values exceeding 300. We produced a cloudogram to visualise the posterior distribution of trees using DensiTree (Bouckaert 2010). A maximum‐clade‐credibility tree with mean heights and 95% HPD intervals for divergence times and posterior node support values was calculated using TreeAnnotator from BEAST (Bouckaert et al. 2019).

The resulting tree was visualised in FigTree (http://tree.bio.ed.ac.uk/software/figtree/). Glacial stages were graphically added to the figure from 0.959 million years onwards, according to the marine isotope stages (MIS) presented in Sun et al. (2019). We adjusted the Saalian glaciation complex to span 0.13–0.3 mya (MIS 6‐MIS 8), as proposed for Northern Central Europe (Lang et al. 2018; Litt et al. 2007). We further evaluated whether divergence occurred more frequently during glacial stages than expected by chance using a chi‐squared test. Specifically, we counted mean divergence time estimated by SNAPPER that fell within glacial stages and assessed whether they were enriched compared to the expected distribution based on the proportion of cooling stages over the past 0.959 mya. Splits dated after the last glacial stage 0.014 mya were excluded, as these populations may not have diverged substantially.

To detect historic gene flow between lineages, we used *D*suite (Malinsky et al. 2021) to calculate ABBA‐BABA statistics amongst individuals, inferring excess allele sharing between taxa. The ABBA‐BABA test (also known as the *D*‐statistic) assesses deviations in allele patterns that would be expected under a simple bifurcating tree model, thereby providing insight into historical admixture events. We used *DsuiteDtrios* on the non‐LD pruned SNPs of the 40 individuals representing the five non‐admixed evolutionary lineages with default options, specifying *S. acaulis* subsp. *cenisia* samples as outgroup. We used ‘plot_d.rb’ (https://github.com/mmatschiner) to plot the most significant *D*‐statistic per pair.

### 2.6 Adaptive Divergence Amongst Genetic Groups in the Alpine Arc

We investigated adaptive genetic variation amongst partly sympatric genetic groups across the European Alpine Arc to assess the role of ecotype formation as a driver of divergence in the *S. acaulis* species complex. To investigate adaptive differentiation related to soil and bedrock characteristics as well as climatic variables, we used partial redundancy analysis (pRDA), which uses a set of environmental variables (i.e., explanatory variables) to explain variation in genetic data (i.e., response variables) while accounting for effects of neutral genetic structure (i.e., conditional variables). To characterise soil properties, we compiled soil predictors from multiple sources: the European Soil Data Centre (ESDAC; Panagos et al. 2012), SoilGrids (Hengl et al. 2014) and two lithological layers from Chauvier et al. (2021), which are based on the Global Lithological Map (GliM; Hartmann and Moosdorf 2012) and the International Hydrogeological Map of Europe (IHME1500; Duscher et al. 2015). Following Chauvier et al. (2021), we selected ESDAC predictors with full spatial coverage and ecological relevance for plants, including data from the 3D Soil Hydraulic Database of Europe (Tóth et al. 2017), the European Soil Database (Hiederer 2013a, 2013b), and Topsoil Organic Content for Europe (Jones et al. 2005). From SoilGrids, we included pH, carbon density and cation exchange capacity. Together, ESDAC and SoilGrids provided a comprehensive set of soil properties. We calculated profile averages for ESDAC variables where both topsoil and subsoil data were available. We acknowledge some uncertainty in our soil data because fine‐scale edaphic variation may not be fully captured, and datasets from different sources can vary in soil characterisation. As climatic predictors, we extracted 19 bioclimatic variables for the reference period between 1981 and 2010 from the CHELSA V2.1 database (Karger et al. 2017). Of the 19 bioclimatic variables, we excluded eight that were calculated on a quarterly basis (e.g., mean temperature of the driest quarter), as the definition of these quarters varied across populations and thus did not allow for consistent comparisons across the study area. Environmental variables were extracted using the *extract* function from the terra R‐package (Hijmans et al. 2022) with bilinear interpolation for numeric descriptors and simple extraction for categorical variables. To account for multicollinearity in the pRDA model, we calculated pairwise Pearson's correlation coefficient (*r*) amongst variables and only retained variables with |*r* | < 0.7 for the analyses (Dormann et al. 2013). More integrative variables were retained over others (e.g., annual mean temperature over minimum temperature of the coldest month or total available water capacity over sand content), as they provide a broader representation of overall environmental conditions. As response variables, we calculated allele frequencies using the complete SNP set and 41 populations with five or more individuals from the European Alps, including admixed populations (*ad*). Missing data were imputed using the mean genotype prior to allele frequency calculation. The first two principal components of a PCA based on allele frequencies were used as conditional variables. We then performed pRDA using the *rda* function from the vegan R‐package (Oksanen et al. 2013) and checked the variance inflation factor (VIF) of the explanatory variables. If explanatory variables exceeded a VIF of >10, the variable with the highest VIF was excluded, and the pRDA was re‐run to further avoid collinearity in the model. We assessed the significance of the global pRDA model, the variance explained by individual pRDA axes, and each explanatory variable using permutation tests implemented via the *anova.cca* function from the vegan package in R. We acknowledge the limited genetic resolution inherent to ddRADseq and therefore use pRDA to visualise genetic associations with environmental gradients in ordination space, focusing on identifying environmentally divergent gene pools rather than on individual adaptive SNPs.

## 3 Results

### 3.1 De Novo Genome Assembly and Annotation

The high quality de novo assembled reference genome for a female *S. acaulis* was based on a single haplotype assembly (eth_SilAcau_F_GE_1.1; www.ncbi.nlm.nih.gov: PRJNA1267970) and had a total size of 1,389,309,622 base pairs (bp) arranged in 12 chromosomes and 2289 small scaffolds with an N50 of 100,665,385 bp. The scaffold N90 was 88,314,192 bp and scaffold L90 was 12, which corresponds to the haploid chromosome number of *S. acaulis* (*n* = 12, Jones and Richards 1962) and indicates a chromosome‐level assembly. BUSCO analysis identified 95.4% complete genes, with single copy orthologs present at 88.9% and duplicated orthologs at 6.5% (BUSCO; eudicots_odb10, *n* = 2326; Manni et al. 2021). The 12 largest scaffolds, representing the 12 chromosomes and covering 1,259,806,463 bp (90.7%), were used for mapping short reads in all subsequent analyses. More detailed results on assembly and annotation can be found in Tables S3 and S4 and Figures S2 and S3.

### 3.2 Genetic Structure and Evolutionary Lineages

**FIGURE 2:**
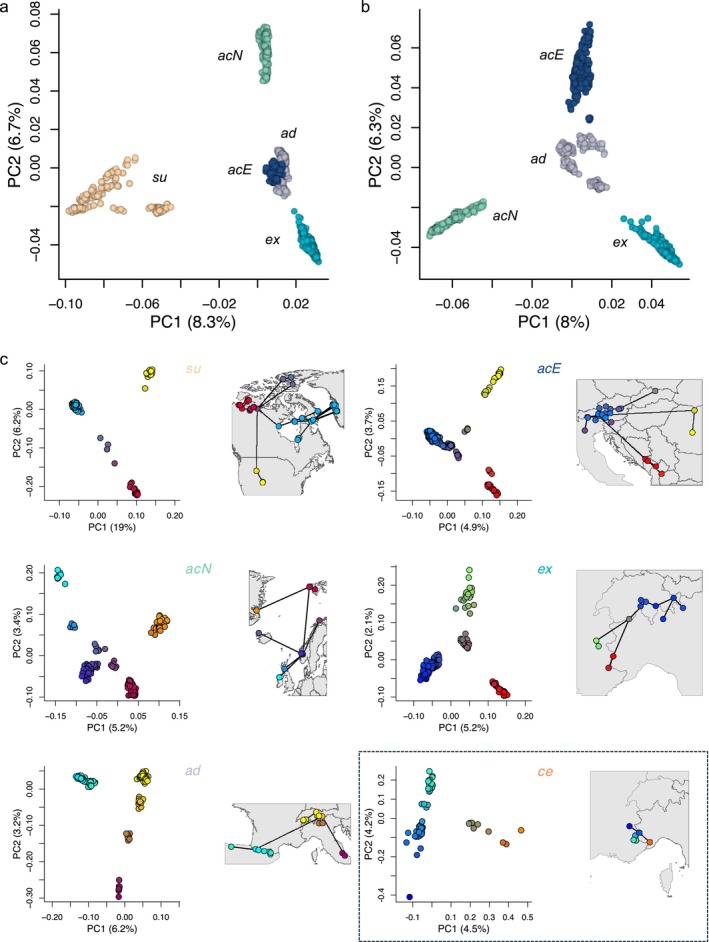
Genetic structure and spatial genetic differentiation within the *Silene acaulis* species complex and its genetic groups. (a) Principal component analysis (PCA) illustrating the genetic structure amongst evolutionary lineages and the admixed group, using the core SNP set (70,827 SNPs), which excludes *S. acaulis* subsp. *cenisia* (*ce*). The proportion of variance explained by each axis is provided in parentheses. (b) PCA depicting the genetic structure amongst evolutionary lineages and the admixed group, excluding both *ce* and *S. acaulis* subsp. *subacaulescens* (*su*). (c) For each genetic group, the left panel shows a PCA plot displaying the first two principal components (PC1 and PC2) based on the core SNP set (except for *ce*). Individuals are colour‐coded based on their positions along the first two PCs. The right panels show the geographic origin of populations within each genetic group, with lines connecting populations based on a minimum spanning tree constructed from pairwise *F*_ST_ values. Population colours on the maps reflect the mean principal component coordinates of each population. Colours and abbreviations are consistent with Figure 1.

The analysis of genetic structure, was conducted using 955 samples and the complete SNP set of 28,666 SNPs. We identified *K* = 5 genetic clusters that are likely to represent the main evolutionary lineages (Figure 1a,b), as suggested by the plateauing of log likelihood values and cross‐validation errors beyond *K* = 5 (Figure S4). Individuals identified a priori as *S. acaulis* subsp. *cenisia* (Vierh., hereafter referred to as *ce*) separated at *K* = 2 (Figure S5) and exhibited the highest genetic differentiation to all other groups (*F*_ST_ range: 0.56–0.69, Figure 1c). At *K* = 5, individuals that were a priori identified as North American *S. acaulis* subsp. *subacaulescens* (N. F. Williams) Hultén (*su*) and dioecious *S. acaulis* subsp. *exscapa* (All.) Braun Blanq. (*ex*) formed separate groups. However, individuals a priori identified as *S. acaulis* subsp. *acaulis* (L.) Jacq. formed three genetically distinct groups. The Northern European samples (hereafter referred to as *S. acaulis* subsp. *acaulis* North, *acN*) and Eastern European samples (hereafter referred to as *S. acaulis* subsp. *acaulis* East, *acE*; Figure 1) formed separate evolutionary lineages. The third group comprised individuals from the Central Alps in Switzerland and Italy, the Central Apennines, the Pyrenees and the Cantabrian Mountains and were genetically admixed. Hereafter, this group will be referred to as the *S. acaulis* admixed group (*ad*). Most *ad* individuals showed a three‐fold admixture signal from *ex*, *acN*, and *acE*. The extent of admixture and the ancestral proportions within *ad* differed between individuals from the Alps, Apennine and Pyrenees/Cantabrian Mountains. The individual for which the reference genome (eth_SilAcau_F_GE_1.1) was assembled is part of the *ad* group from the Swiss Alps. Pairwise *F*_ST_ values between the admixed group (*ad*) and all other groups ranged from 0.09 to 0.26, indicating considerable historical divergence rather than recent hybridization. The different hierarchical PCAs (Figures 1e and 2a,b) confirmed the general pattern of genetic structure as identified by ADMIXTURE and revealed more detailed substructure. The first principal component explained 19.3% of genetic variation, strongly separating *ce* from all other lineages (Figure 1e). Interestingly, when samples belonging to *ce* and *su* were excluded from the PCA, *ad* formed separate and intermediate groups (Figure 2b). Observed heterozygosity (*H*_O_) differed significantly between all evolutionary lineages and the admixed group, except between *ex* and *ad* (Table S5). Overall, the highest levels of *H*_O_ were observed in *ad*, *ex* and *acE*, followed by *acN*, *su* and *ce* (Figure 1d and Figure S6).

We investigated the genetic structure and genetic differentiation within each of the five evolutionary lineages and the admixed group using PCAs and minimum spanning trees based on the core SNP set comprising 70,827 SNPs and 892 individuals (Figure 2c), except for *ce*, where we used the complete SNP set with 28,666 SNPs. The PCAs revealed strong genetic substructure within each lineage (Figure 2). The minimum spanning trees based on *F*_ST_ revealed potential (re‐)colonisation patterns of populations within evolutionary lineages and the admixed group.

### 3.3 Phylogenetic Relationships and Introgression

**FIGURE 3:**
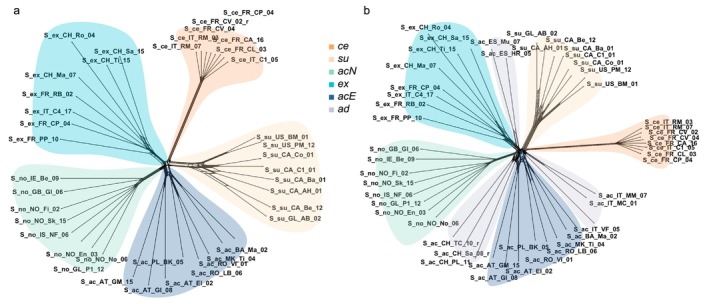
Unrooted Neighbour Net networks visualising genetic relationships in the *Silene acaulis* species complex. (a) Network based on 40 representative individuals and 5176 LD‐pruned SNPs, excluding the admixed group (*ad*). (b) Network based on 48 individuals and 5036 LD‐pruned SNPs, including the admixed group (*ad*). Colours and abbreviations are consistent with Figure 1.

The phylogenetic network based on a representative subset consisting of 40 individuals (Figure S7) and 5176 LD‐pruned SNPs revealed a clear divergence of *S. acaulis* subsp. *cenisia* (*ce*) from the remaining lineages, which were more closely clustered and showed signs of reticulate evolution (Figure 3a). Populations identified as belonging to the same lineage consistently clustered together in the phylogenetic network. When admixed populations (*ad*) were included in the analysis (48 individuals and 5036 LD‐pruned SNPs), they did not form one distinct lineage. Instead, they occupied intermediate positions within the network, showing reticulate patterns and clustering according to their geographic origin (Figure 3b).

**FIGURE 4:**
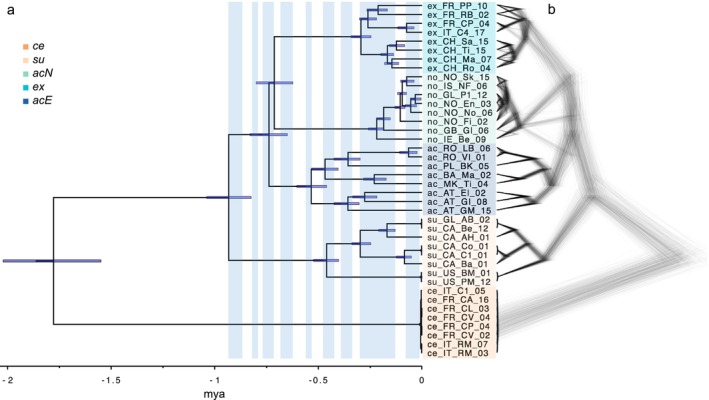
Phylogenetic relationships amongst evolutionary lineages of the *Silene acaulis* species complex inferred using SNAPPER in BEAST. (a) Dated phylogenetic tree, with glacial phases up to 0.936 million years ago (mya) highlighted in light blue. Glacial cycles were inferred from marine isotope stages (Sun et al. 2019). (b) Cloudogram representing the posterior distribution of BEAST trees, showing phylogenetic uncertainty and support for evolutionary relationships. Colours and abbreviations are consistent with Figure 1.

Phylogenetic tree inference based on SNAPPER in BEAST and 5176 LD‐pruned SNPs (excluding *ad*) confirmed the five evolutionary lineages identified with ADMIXTURE as monophyletic groups (Figure 4, Table S6). However, the phylogenetic relationships amongst the Northern lineage (*acN*), *S. acaulis* subsp. *exscapa* (*ex*), and the Eastern lineage (*acE*) were uncertain, as reflected in the posterior distribution of trees and low posterior support values for the node connecting *ex* and *acN* (Figure S8). This could suggest a trichotomy amongst these lineages, prompting further investigation into whether reticulation could account for this uncertainty. Within each lineage, we observed overall well‐resolved patterns of divergence, confirming the mostly pronounced population structure observed in the within‐lineage PCAs (Figure 2). The least well‐resolved phylogenetic relationships within lineages were found to be the *acN*, *ex*, and *S. acaulis* subsp. *cenisia* (*ce*), where posterior trees and support values are compatible with incomplete lineage sorting (Figure 4b and Figure S8).

Divergence time estimation dated the first split separating the European *ce* from the rest of the species complex to 1.78 (1.55–2.02) million years ago (mya), followed by the divergence of the North American *S. acaulis* subsp. *subacaulescens* (*su*) from *ex*, *acN*, and *acE* 0.93 (0.82–1.04) mya (Figure 4a). The remaining three lineages (*ex*, *acN* and *acE*) diverged at a similar time, around 0.71–0.74 (0.62–0.83) mya. The timing of further splits and diversifications within evolutionary lineages varied substantially between lineages: *acE*: 0.53 (0.46–0.60) mya; *su*: 0.46 (0.40–0.52) mya; *ex*: 0.29 (0.25–0.34) mya; *acN*: 0.22 (0.18–0.26) mya; *ce*: 0.0065 (0.0029–0.013) mya. Interestingly, 79% of the splits in the phylogeny occurred during glacial periods, which is significantly more than would be expected by chance (Χ^2^ = 5.35, *p* = 0.020).

**FIGURE 5:**
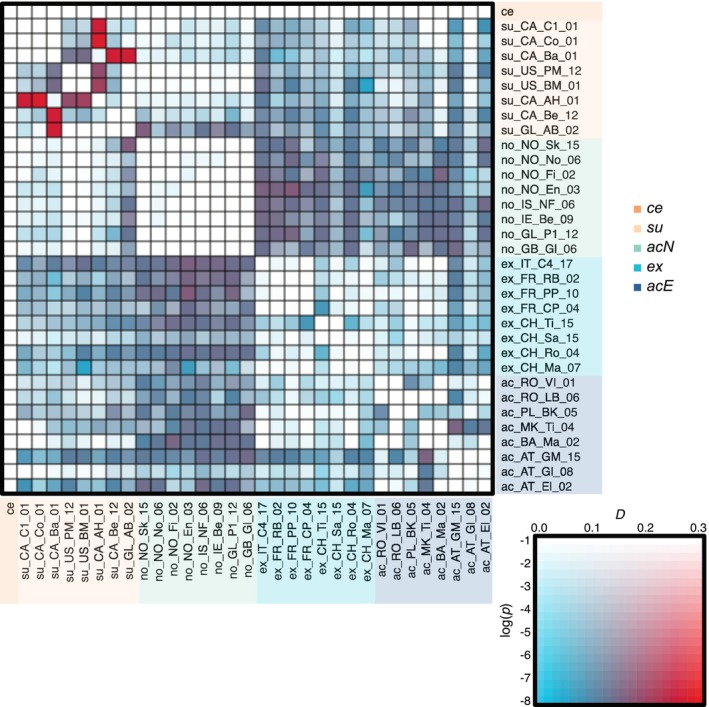
*D*‐statistics, that is, historic gene flow, inferred with *D*suite based on the five evolutionary lineages of the *Silene acaulis* species complex. The most significant *D*‐statistics values for all possible trios of populations are plotted with magnitude coloured from blue to red and statistical significance from light to dark, as indicated in the legend. Colours and abbreviations are consistent with Figure 1.

*D*‐statistics (ABBA‐BABA tests) identified relatively high levels of historic gene flow between all populations of *acN* and *ex*, as well as between *acN* and *acE* (Figure 5). Similar but less pronounced signals were found between all populations of *su* and *ex*, as well as between *su* and *acE*. The consistent pairwise signals of introgression amongst populations within a lineage suggest that gene flow likely occurred before within‐lineage divergence. Additionally, some individual populations exhibited excessive allele sharing both within and between lineages. The highest levels of historic gene flow were found between a Central Canadian population (su_CA_Ba) and populations in Eastern Canada (su_CA_Be) and Western Greenland (su_GL_AB).

### 3.4 Adaptive Divergence Amongst Genetic Groups in the Alpine Arc

**FIGURE 6:**
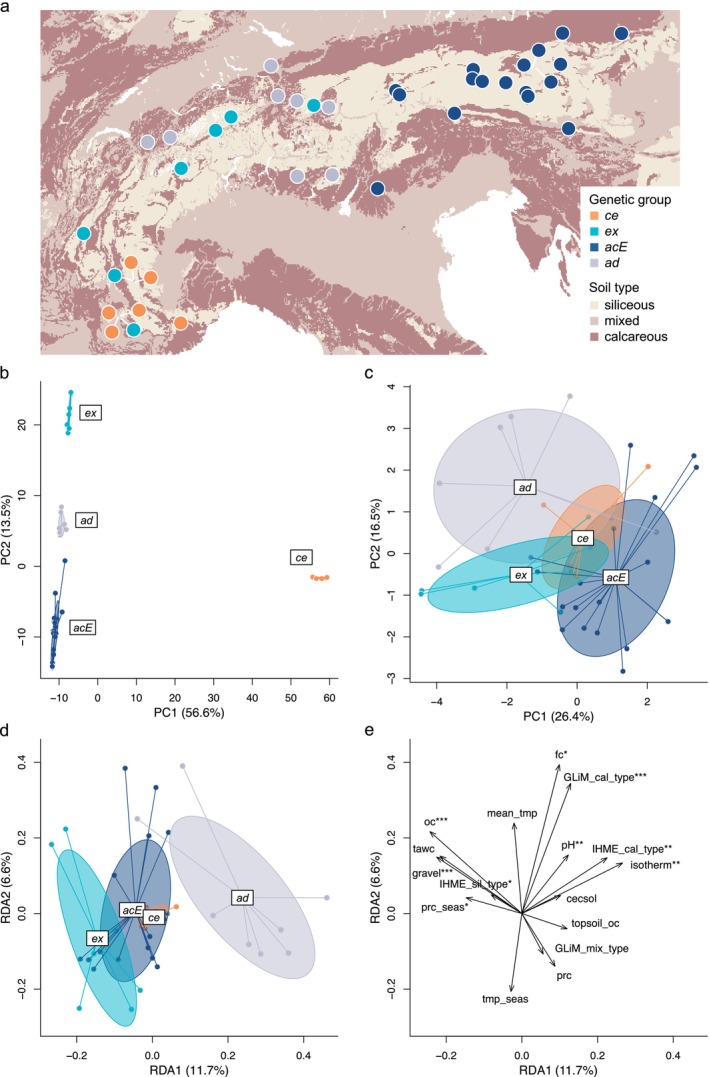
Adaptive divergence amongst four partly sympatric genetic groups of the *Silene acaulis* species complex in the European Alps. (a) Map of the Alpine Arc showing population origins, with background coloured by soil lithology (GliM bedrock type layer: Chauvier et al. 2021). (b) Neutral genetic structure inferred from a principal component analysis (PCA) of population allele frequencies based on 28,666 SNPs, with variance explained by each axis in parentheses. (c) Environmental niches inferred from PCA based of 16 uncorrelated environmental variables. (d) Adaptive genetic composition from partial redundancy analysis (pRDA) of genetic variation conditioned on neutral genetic structure and constrained by the same 16 environmental variables. (e) Biplot of the pRDA illustrating the relative contribution of environmental variables to the genetic variation (variable abbreviations correspond to those in Table S7). Significance of each variable's contribution was assessed via permutation tests (*p < 0.05, **p < 0.01, ***p < 0.001). Colours and abbreviations of genetic groups are consistent with Figure 1.

To explore ecological differences amongst the three evolutionary lineages as well as the admixed group in the Alpine Arc, we analysed population allele frequencies of 28,666 SNPs and 16 uncorrelated environmental variables across 41 populations representing *ce*, *ex*, *acE*, and *ad* (Figure 6a, Tables S7–S9). The genetic groups differed in their overall neutral genetic composition (Figure 6b, Figure S9) and to some degree in their environmental niche (Figure 6c, Figure S9), as inferred with separate PCAs. Partial redundancy analysis (pRDA) was used to evaluate the contribution of environmental variables to genetic variation, while controlling for neutral genetic structure. The global pRDA model explained a significant portion of the genetic variation (permutation test: *F* = 1.5, *p* = 0.001). Permutation tests for individual constrained axes were significant for RDA1 (*F* = 5.5, *p* = 0.001) and RDA2 (*F* = 3.1, *p* = 0.001). Seven soil variables and two climate variables contributed significantly to the pRDA model (Figure 6d,e, Table S10). The pRDA indicated that 30.5% of the genetic variation could be explained by environmental variables while accounting for the effect of neutral genetic structure, which explained 42.1% of genetic variation (Figure 6, Table S10). The pRDA revealed that individuals from different genetic groups occupied distinct positions along the first two constrained axes (Figure 6d). Populations belonging to *acE* clustered close to the centre of the pRDA space of the first two axes, which in sum explained 18.3% of genetic variation after removing the contribution of neutral genetic structure. Partially sympatric populations of *ad* and *ex* were found to be differentiated on the first two axes, with soil type and isothermality (i.e., the ratio of daily to annual temperature variation) being the main differentiating factors (Figure 6e). Adaptive genetic composition of *ad* populations was mainly explained by calcareous soil type and increasing isothermality. On the other hand, populations of *ex* clustered partly overlapping with *acE*, with genetic variation explained by non‐calcareous soils and decreasing isothermality. Within‐group genetic variation was mostly explained by organic carbon content and gravel content.

## 4 Discussion

Our findings indicate that, within the scope of our Holarctic sampling, the *Silene acaulis* species complex comprises five major evolutionary lineages and an admixed group, with the Alps representing the centre of diversification. Evolutionary lineages primarily diverged within glacial refugia during Pleistocene cooling phases. Ecological divergence and niche specialisation between sympatric genetic groups has further contributed to shaping the observed genetic and spatial structure, driving diversification in particular amongst genetic groups in the Cantral and Western Alps. Adaptation to distinct environmental conditions or genetic drift in refugia, coupled with the reshuffling of genetic diversity during periods of secondary contact, has likely fueled soil‐specific ecotypic diversity and sexual system divergence in *S. acaulis* . High intraspecific variation, shaped by repeated cycles of allopatry and secondary contact in refugia, may have contributed to the ability of *S. acaulis* to repeatedly shift its range in response to past climatic changes (Crawford 2008).

### 4.1 Evolutionary Lineages

The divergence of the evolutionary lineages is evidenced by relatively high pairwise *F*_ST_‐values and their monophyly. The varying levels of genetic diversity across lineages likely reflect differences in recolonization dynamics, the size of refugial areas, and admixture history. Notably, our genetic data revealed significant genetic substructure within *S. acaulis* subsp. *acaulis*, aligning with genetic borders identified for many arctic‐alpine plant species across the Greenlandic ice cap and between the European Alps and the Arctic (Eidesen et al. 2013). Focused sampling across central European mountain ranges revealed substantial intraspecific diversity with four distinct groups, including *S. acaulis* subsp. *cenisia* (*ce*), *S. acaulis* subsp. *exscapa* (*ex*), and *acE*, alongside admixed populations (*ad*) distributed across the Pyrenees, Cantabrian mountains, Central Alps, and Central Apennines. Admixed populations (*ad*) exhibit relatively homogeneous ancestry coefficients within each geographic area and relatively high divergence to other evolutionary lineages (*F*_ST_ 0.09–0.56), suggesting that admixture may have occurred many generations ago, likely when the three evolutionary lineages *ex*, *acE*, and *acN* came into secondary contact during one or several Pleistocene glacial cycles. These findings reveal a more complex glacial history of the *S. acaulis* complex in Europe than previously recognised and provide a comprehensive characterisation of the intraspecific diversity of *S. acaulis* in Europe. High observed genetic diversity (*H*_O_) in *acE*, *ex*, and *ad*, relative to *acN* and *su*, except for *ce*, supports the Alpine Arc as the origin of evolutionary lineages included in this study, which was also proposed by Gussarova et al. (2015 and references therein). Support for this ‘out of the Alps hypothesis’ has been found in other arctic‐alpine species, such as in *Ranunculus glacialis* , which has colonised northern Europe from source populations in the eastern Alps (Schönswetter et al. 2003). Interestingly, the genetic structure identified for *S. acaulis* broadly corresponds with that documented for a specialised pathogen, the anther‐smut fungus *Microbotryum silenes‐acaulis* (Bueker et al. 2016). It is tempting to speculate that the increased prevalence of the pathogen in more northern latitudes could be linked to the lower levels of genetic diversity found in its host.

### 4.2 Phylogenetic Dating and Glacial Cycles

**FIGURE 7:**
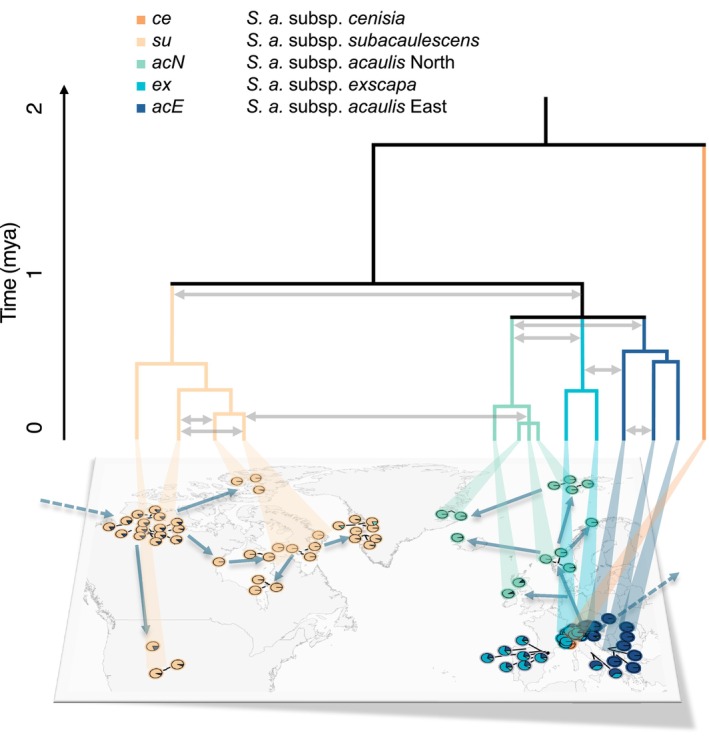
Schematic representation of the evolutionary history of the *Silene acaulis* species complex. Divergence times are based on SNAPPER estimates. Grey arrows schematically indicate major historic gene flow events between lineages and populations as inferred using *D*suite. Blue arrows represent colonisation pathways, qualitatively reconstructed from spatial genetic differentiation patterns. The timing of the hybrid origin of the admixed group (*ad*) remains uncertain and is therefore not depicted.

Our study shows that the diversification of the *S. acaulis* species complex is tightly linked to climatic oscillations during the Pleistocene. While acknowledging potential uncertainties associated with secondary calibration, the estimated timing of most lineage splits aligns with glacial periods. Our molecular data indicate that the first split, separating *S. acaulis* subsp. *cenisia* (*ce*) from the other evolutionary lineages, occurred 1.78 mya, which coincided with the onset of the Middle Pleistocene (1.8 mya; Head and Gibbard 2005). The early divergence, together with the recent and limited divergence between populations of *ce* after the last glacial maximum (LGM), may reflect long‐term isolation and an initially restricted distribution range, as further supported by the low levels of observed heterozygosity. Additionally, the restricted distribution of *ce* in the Southwestern Alps, a region known to harbour numerous paleoendemics such as *Saxifraga florentula*, further supports the role of this area as a refugium for cold‐adapted flora throughout the Pleistocene (Parisod 2022). After the split of *su* from the other lineages 0.93 mya, suggesting a single colonisation of North America and Western Greenland, the other main lineages diverged around 0.71–0.74 mya during the Early‐Middle Pleistocene transition (0.78 mya; Head and Gibbard 2005). Within evolutionary lineages, genetic variation and phylogenetic structure often reflect isolation by distance, revealing potential recolonization routes (Figure 7). Divergence within the main extant lineages occurred primarily during multiple advances of the ice sheets during the Elsterian glaciations (0.48–0.42 mya and 0.37–0.34 mya) and the powerful Saalian glaciation complex (0.3–0.13 mya), where vast areas of the Northern hemisphere were covered by deep ice‐sheets (Bandou et al. 2022; Ehlers and Gibbard 2008; Lang et al. 2018). While the main lineages diverged throughout the Pleistocene, the within lineage diversification in the Northeast Atlantic region was relatively recent, primarily shaped by the last two glacial cycles. This aligns with the findings of Gussarova et al. (2015) who proposed that Northern populations have a relatively recent history, influenced by long‐distance dispersal events. Northern *S. acaulis* populations (*acN*) may have recolonized the northern amphi‐Atlantic area from few refugia south of the large ice sheets covering the Northern Hemisphere during the last glacial maximum, as has been inferred for other plant species (Brožová et al. 2023; Eidesen et al. 2013; Koch et al. 2006; Winkler et al. 2012). On Greenland, the presence of the Northern lineage (*acN*) in the east and the North American lineage (*su*) in the west suggests two independent colonisation events.

The majority of inferred phylogenetic splits (79%) likely occurred during glacial cooling phases during the last one million years. Similar findings of phylogenetic splits synchronised with glacial cycles have been reported in brown bears (da Silva Coelho et al. 2023) and ginkgo (Hohmann et al. 2018). *Silene acaulis* is an arctic‐alpine species whose distribution was most likely restricted to areas close to the glaciers and isolated ice‐free mountain tops during cooling phases (Holderegger and Thiel‐Egenter 2009; Schönswetter et al. 2003). Given these ecological constraints, we hypothesize that population divergence in *S. acaulis* was primarily driven by isolation in allopatric refugia during glacial periods, rather than by range expansion. This interpretation aligns with well‐documented patterns in other arctic‐alpine species, where glacial cooling phases forced populations into fragmented habitats, limiting gene flow and promoting genetic divergence through drift or local adaptation (Kadereit 2023; Stewart et al. 2010).

### 4.3 Secondary Contact, Hybridization and Introgression

Recurring phases of isolation and secondary contact during glacial cycles provided ample opportunities for hybridization amongst populations of arctic‐alpine plant species (Parisod 2022). In line with this scenario, we found signals of excessive allele sharing between northern amphi‐atlantic populations (*acN*) and the western Alpine *ex* lineage, as well as between *acN* and *acE*, indicative for historic gene flow between lineages during some of the glacial/interglacial periods. Secondary contact potentially occurred between 0.5–0.7 mya, predating the divergence of populations within lineages, as most populations of the lineages show elevated *D*‐statistics amongst *acN, acE* and *ex* (Figure 7). The inference of gene flow amongst these lineages is further supported by less well resolved splits in the phylogeny. These signatures of secondary contact may indicate a complex scenario involving gene flow events between all three lineages. Excessive allele sharing between *acN* and the other European lineages, as well as *acN* ancestry found in the admixed group (*ad*), suggest that *acN* survived in Central European refugia while the Northern hemisphere was covered by ice sheets. The idea of a Central European refugium for *acN* prior to its expansion is further supported by the relatively recent origin (0.22 mya) of the Northern lineage. Hence, hybridization during secondary contact, potentially accompanied by extinction events and long‐distance dispersal appear to have significantly impacted the early evolutionary history of the three sister lineages *acE*, *ex* and *acN* and likely even the North American *su* lineage. This is also supported by the existence of populations with admixed ancestry (*ad*) that appear to be in the process of diverging into evolutionary lineages, as indicated by their homogenous admixture proportions and moderately high divergence from other lineages. The continuous reshuffling of genetic variation during secondary contact likely enhanced the adaptive potential and facilitated adaptive diversification in response to dynamic environmental conditions (Abbott et al. 2013; Maier et al. 2019; Marques, Lucek, et al. 2019; Marques, Meier, and Seehausen 2019).

The introgression analysis also indicates excessive allele sharing between populations within the evolutionary lineage from Northern and Eastern Canada, which have diverged from each other up to 0.3 mya. This suggests common refugia for these populations in Northern Canada, further supported by the minimum spanning tree based on *F*_ST_‐values. Alternatively, these patterns of historic gene flow amongst North American populations could indicate the presence of refugial areas in Beringia. Beringia served as an important area for initial dispersal and long‐term survival of *Vaccinium uliginosum* (Alsos et al. 2005) and many other arctic species (Eidesen et al. 2013; Skrede et al. 2009). A schematic illustration of the complex evolutionary history of *Silene acaulis* is presented in Figure 7.

### 4.4 Adaptive and Sexual System Divergence in Sympatry

A sharp genetic transition at the border between the Western and Eastern Alps, corresponding to a well‐established phylogeographic divide (Schönswetter et al. 2005), separates the Eastern (*acE*) from the Western *S. acaulis* genetic groups (*ex*, *ce*, and *ad*). Hence, the Western and Central Alps host sympatric populations of *ex*, *ce*, and *ad*, raising the question about their divergence. The central position of *acE* along the first two pRDA axes suggests that *acE* may represent a generalist ecotype, and populations of this lineage can indeed grow on both calcareous and siliceous bedrock (pers. comm. A.T.). In contrast, genetic variation in *ad* and *ex* shows stronger associations with specific environmental variables and these groups differ most strongly in their associations with soil characteristics, thereby partitioning the adaptive niche space. Furthermore, *ex* appears to be adaptively differentiated from the sympatric and older *ce* lineage in the Western Alps. These findings suggest that in the Western and Central Alps the genetic groups occur sympatrically but grow parapatrically in different edaphic and climatic niches, aligning with patterns observed in other Alpine plants. In *Senecio carniolicus*, for example, ecological differentiation between cytotypes was found to be stronger in areas of sympatry, suggesting that co‐occurrence can reinforce ecological divergence (Sonnleitner et al. 2016).

Differences in edaphic preferences between *S. acaulis* subsp. *exscapa* (*ex*, siliceous bedrock) and subsp. *acaulis* in Switzerland (*ad*, calcareous bedrock) have long been noted (Aeschimann et al. 2004). Our results demonstrate that the two taxa are genetically differentiated and strongly associated with their respective soil environments. Genetic variation in these genetic groups is associated with different edaphic and climatic factors, indicating that their divergence has a strong adaptive component. Such divergence may have evolved in distinct glacial refugia, where adaptation to different soils and climatic conditions proceeded simultaneously. This interpretation is consistent with broader biogeographic patterns identified for the Alpine flora. Silicicolous species have persisted predominantly in nunatak refugia during the LGM, where rugged topography and siliceous substrates jointly provided stable habitat conditions (Rosa et al. 2025). Such conditions match the adaptive profile of *ex*, which was likely restricted to nunataks during the LGM. As nunataks were overwhelmingly siliceous and thus unsuitable for calcareous plants, *ad* likely persisted in peripheral or extra‐alpine refugia, where calcareous substrates remained exposed (Rosa et al. 2025). The contrasting microclimatic regimes of siliceous versus calcareous soils may therefore have acted as persistent selective pressures during refugial periods. Taken together, the results suggest that the observed adaptive patterns likely reflect ecotypic differentiation shaped by long‐term selection mediated by lithology, topography, and microclimate in refugia. Furthermore, *ex* is the only dioecious lineage (Maurice et al. 1998) and co‐occurs with two trioecious groups, *ce* and *ad*. This pattern may reflect additional reinforcement of ecological differences through sexual system divergence. Interestingly, we find that the evolution of dioecy in *ex* is likely very recent and may have evolved between 0.74 and 0.29 million years ago.

### 4.5 Outlook

As current climate change exerts substantial pressures on arctic‐alpine ecosystems, understanding their responses to past climatic changes is of great interest for predicting future adaptability. *Silene acaulis*, as a facilitator, nurse plant, and foundation species, plays a key role in arctic‐alpine ecosystems (Alatalo and Little 2014; Molenda et al. 2012). However, with *S. acaulis* projected to experience significant range contractions due to climate change (Wessely et al. 2022), the persistence of many populations is at risk, and shrinking habitats may result in rapid population declines and an associated loss of genetic diversity. Given its ecological importance, climate sensitivity, and high intraspecific diversity in both ecotypes and sexual systems, *S. acaulis* presents an ideal model for investigating the evolutionary and ecological dynamics of arctic‐alpine species in the face of climate change. The newly assembled haplotype‐based reference genome provides a foundational resource for future genomic and functional studies, enabling deeper insights into the genetic basis of adaptation, sexual system evolution, and the complex history of introgression and admixture. The existence of multiple evolutionary lineages that differ in ecological preferences, distribution, and mating system makes it likely that responses to ongoing climate change will also differ. Our results lay the foundation for developing species distribution models that consider genetic groups and adaptive variation for predicting climate change responses. This approach could enhance our understanding of the relevance of diversification driven by glacial cycles and uncover previously unrecognised potential for adaptation to a changing climate.

## Author Contributions

M.C.F. conceived the project. O.R., N.Z., and M.C.F. performed data analyses. G.H.‐N. assembled the reference genome. M.C.F., A.T., and P.T.I. collected data. O.R., M.C.F., and N.Z. contributed to computer code and discussions. A.W. provided conceptual input. O.R. and M.C.F. wrote the manuscript with input from all co‐authors.

## Funding

The authors have nothing to report.

## Disclosure

We carefully assessed the Access and Benefit‐Sharing (ABS) frameworks of all countries where samples were collected and ensured compliance with all relevant regulations. In cases where official information was incomplete or unclear, we directly contacted national authorities to confirm requirements.

## Conflicts of Interest

The authors declare no conflicts of interest.

## Supporting information


**Figure S1:** Histogram showing the distribution of the 955 individuals of the *Silene acaulis* species complex included in the genetic analysis across 132 sampled locations, based on the number of individuals per location. The colours represent the genetic groups to which the individuals belong, with group assignments indicated in the legend. The x‐axis denotes the number of individuals sampled per location, and the y‐axis shows the corresponding number of locations.
**Figure S2:** Genome size and heterozygosity estimation for *Silene acaulis* . The GenomeScope profile shows kmer‐based estimates of genome size (len), heterozygosity (ab) and repeat content (100‐uniq) from the frequency of *k*‐mers (*k* = 21) within HiFi reads.
**Figure S3:** Reference genome assembly evaluation. (a) The assembly k‐mer (k = 21) copy number spectrum shows few errors and low level of duplications within eth_SilAcau_F_GE_1.fa, the reference for this study. (b) The shared k‐mer copy number spectrum shows even distribution of heterozygous k‐mers between the two haplotype assemblies uploaded. Omni‐c contact maps show strong contact frequency along the diagonal within the 12 scaffold groups (leftmost squares) of (c) eth_SilAcau_F_GE_1 and (d) eth_SilAcau_F_GE_2 corresponding to chromosomes. Scaffold groups are ordered in ascending order from left to right; SG_1 to SG_12. Unplaced scaffolds are also depicted.
**Figure S4:** Log‐likelihood values (left) and cross‐validation (CV) errors (right) resulting from ADMIXTURE analysis for *K* = 1–10 ancestral populations with 955 individuals and 28,666 SNPs. Error bars indicate the standard deviation based on 10 repetitions.
**Figure S5:** CLUMPAK output for admixture runs for the ADMIXTURE analysis for *K* = 2–10 ancestral populations using 955 individuals of the Silene acaulis species complex and 28,666 SNPs. Shown are the minor and major modes of the runs and the divisions of minor and major modes. Samples are grouped according to their country of origin.
**Figure S6:** Boxplot illustrating the distribution of observed heterozygosity (H_O_) values across genetic groups. Boxes represent the interquartile range with median values shown as horizontal lines. Mean values are displayed above each box, and letters indicate statistically significant differences between groups based on ANOVA.
**Figure S7:** Representative selection of samples within the different evolutionary lineages of the *Silene acaulis* species complex for the phylogenetic analyses. For each genetic group, a PCA and map with sampling locations coloured according to average PCA values and connected based on a minimum spanning tree is shown. Black circles indicate the genetic clusters from which we selected a certain number of samples. We aimed to select an equal number of individuals per PCA cluster, totaling to eight samples. Clusters represented by less populations were less weighted, if the total number of clusters was not divisible by eight. Samples were selected based on highest coverage and, if possible, different populations were considered.
**Figure S8:** Phylogenetic tree of the *Silene acaulis* species complex inferred using SNAPPER in BEAST, based on 5176 LD‐pruned SNPs from 40 individuals. Posterior node support values are indicated with numbers; the scale is given in units of million years ago (mya).
**Figure S9:** Principal component analyses of genetic and environmental variation. (a) Proportion of variance explained by the first ten principal components derived from a PCA of allele frequencies across 41 populations in the European Alps. Bars indicate the relative contribution of each component to the total genetic variation. (b) PCA biplot based on 16 uncorrelated environmental variables. Arrows indicate the relative strength and direction of each variable's contribution to the principal components (variable abbreviations are provided in Table S7).

**Table S1:** Overview of the 132 populations included in the genetic analysis of the *Silene acaulis* species complex.
**Table S2:** Overview of SNP sets and SNP filtering steps used in this study. MinDP: minimum depth; minQ: minimum quality; maxmis: missing data threshold (0 = all missing allowed; 1 = no missing allowed); mac: minor allele count; minmeanDP: minimum mean depth; MaxDP: maximum depth.
**Table S3:** Assembly statistics for both haplotype assemblies. BUSCO score shows complete (C), single copy (S), duplicated (D), fragmented (F), and missing (M) genes discovered in the assembly sequence compared to the total number of reference genes in the eudicots ODBv10 dataset (n); run in genome mode.
**Table S4:** Annotation statistics for gene predictions of both haplotype assemblies. BUSCO score shows complete (C), single copy (S), duplicated (D), fragmented (F), and missing (M) translated sequences from the gene models compared to the total number of reference genes in the eudicots ODBv10 dataset (n); run in protein mode.
**Table S5:** Pairwise comparisons of observed heterozygosity (HO) amongst the six genetic groups of the *Silene acaulis* species complex. Shown are the Tukey's HSD test results based on ANOVA. The table includes the difference in means, 95% confidence intervals, and adjusted *p*‐values for each comparison.
**Table S6:** Summary of posterior distributions for main parameters of the Bayesian species tree inference using SNAPPER in BEAST. The table presents the mean, 95% highest posterior density (HPD) intervals, and effective sample size (ESS) for each parameter.
**Table S7:** Initial set of environmental descriptors used to investigate adaptive divergence in the European Alps.
**Table S8:** Pairwise Pearson's correlation coefficients based on the initial set of environmental variables of 41 populations used for the investigation of adaptive divergence in the European Alps. Red indicates correlations with |*r*| > 0.7. Variables in bold were retained in the final models.
**Table S9:** Overview of populations of the *Silene acaulis* species complex included in the investigation of adaptive divergence in the European Alps and corresponding geographic coordinates, PCA scores and environmental data for 16 uncorrelated variables included in the analysis.
**Table S10:** mec70254‐sup‐0002‐Tables.xlsx. *F*‐ and *p*‐values from permutation tests evaluating the significance of predictors in the partial redundancy analysis (pRDA). Biplot scores of environmental variables for each pRDA axis, along with the proportion of variance explained after accounting for neutral genetic structure.

## Data Availability

The *Silene acaulis* reference genome eth_SilAcau_F_GE_1 (and the corresponding raw reads) can be found at NCBI (www.ncbi.nlm.nih.gov) under PRJNA1267970. ddRAD raw reads can be found at NCBI under PRJNA1268723. VCF‐files, phylogenetic tree files, and functional annotations of the reference genome are available at the Dryad digital repository: https://doi.org/10.5061/dryad.xgxd254ts.
