# Supplementary material for: The Impact of Pleistocene Glacial Cycles on the Evolutionary Diversification of the Arctic‐Alpine Silene acaulis Species Complex
Source: Mol Ecol. 2026 Jan 23;35(2):e70254. doi: 10.1111/mec.70254 (PMC12831043; doi:10.1111/mec.70254)
Supplement: Supplementary file 1 — Figure S1: Histogram showing the distribution of the 955 individuals of the Silene acaulis species complex included in the genetic analysis across 132 sampled locations, based on the number of individuals per location. The colours represent the genetic groups to which the individuals belong, with group assignments indicated in the legend. The x‐axis denotes the number of individuals sampled per location, and the y‐axis shows the corresponding number of locations. Figure S2: Genome size and heterozygosity estimation for Silene acaulis . The GenomeScope profile shows kmer‐based estimates of genome size (len), heterozygosity (ab) and repeat content (100‐uniq) from the frequency of k‐mers (k = 21) within HiFi reads. Figure S3: Reference genome assembly evaluation. (a) The assembly k‐mer (k = 21) copy number spectrum shows few errors and low level of duplications within eth_SilAcau_F_GE_1.fa, the reference for this study. (b) The shared k‐mer copy number spectrum shows even distribution of heterozygous k‐mers between the two haplotype assemblies uploaded. Omni‐c contact maps show strong contact frequency along the diagonal within the 12 scaffold groups (leftmost squares) of (c) eth_SilAcau_F_GE_1 and (d) eth_SilAcau_F_GE_2 corresponding to chromosomes. Scaffold groups are ordered in ascending order from left to right; SG_1 to SG_12. Unplaced scaffolds are also depicted. Figure S4: Log‐likelihood values (left) and cross‐validation (CV) errors (right) resulting from ADMIXTURE analysis for K = 1–10 ancestral populations with 955 individuals and 28,666 SNPs. Error bars indicate the standard deviation based on 10 repetitions. Figure S5: CLUMPAK output for admixture runs for the ADMIXTURE analysis for K = 2–10 ancestral populations using 955 individuals of the Silene acaulis species complex and 28,666 SNPs. Shown are the minor and major modes of the runs and the divisions of minor and major modes. Samples are grouped according to their country of origin. Figure S6: Boxplot ill [file MEC-35-e70254-s001.pdf]

Supplementary Material and Figures for:

The impact of Pleistocene glacial cycles on the evolutionary diversification of the arctic-alpine *Silene acaulis* species complex

Oliver Reutimann[1†], Gwyneth Halstead-Nussloch[1], Andreas Tribsch[2], Pablo Tejero Ibarra[3], Niklaus Zemp[4], Alex Widmer[1*] & Martin C. Fischer[1†*]

[1] Institute of Integrative Biology, ETH Zurich, Zurich, Switzerland
[2] Department of Environment and Biodiversity, University of Salzburg, Salzburg, Austria
[3] Herbarium JACA, Pyrenean Institute of Ecology-CSIC, Jaca, Spain
[4] Genetic Diversity Centre (GDC), ETH Zurich, Zurich, Switzerland
[*] Corresponding authors: martin.fischer@usys.ethz.ch, martin_c_fischer@icloud.com, alex.widmer@usys.ethz.ch

Supplementary Material

De novo *assembly of the* Silene acaulis *reference genome*

We *de novo* assembled a haplotype phased reference genome (eth_SilAcau_F_GE_1) from a single female *S. acaulis* individual from Switzerland (S_ac_CH_Ge_11; 46.397°N, 7.606°E, see Table S1). A specimen of this individual has been deposited at the United Herbaria Zurich Z+ZT under the number ZT-00296001. High molecular weight DNA was extracted from leaf tissue using Macherey-Nagel NucleoBond HMW DNA kit adapted for plants. DNA was sent to Novogene (Novogene Company Limited, Cambridge, UK) for sequencing on the PacBio Revio platform on a single 25M SMRT cell to produce HiFi reads. Sequencing yielded a total of 102 Gb (6,114,812 HiFi reads, N50 = 16,757 bp), corresponding to approximately 78× coverage of the genome size estimated by GenomeScope2.0 (Ranallo-Benavidez et al., 2020) using the *k*-mer (*k*=21) frequency produced with meryl (Rhie et al., 2020). Snap frozen leaf material was sent to Dovetail Genomics (Cantata Bio, CA, USA) for generation of Omni-C library and deep sequencing. *De novo* haplotype phased assemblies were generated using hifiasm (Cheng et al., 2022) v0.19.8-r603 with HiFi and Omni-C data as input and purge duplications option set to '-l 3'. Omni-C reads were aligned to the haplotype assemblies separately and valid read pairs filtered based on recommended pipeline from Dovetail Genomics. Valid read pairs were then used to scaffold contigs with YaHS (Zhou et al., 2023) and then mapped to the resulting scaffolds for further manual curation in PretextView v0.2.5 (https://github.com/sanger-tol/PretextView) following the Rapid Curation guidelines from Darwin Tree of Life (https://gitlab.com/wtsi-grit/rapid-curation). Assembly metrics were calculated using asmstats (https://github.com/marcelauliano/Teaching/blob/main/asmstats) and seqtk v1.4-r130-dirty (https://github.com/lh3/seqtk). The completeness of the assembly and annotation (see below) was assessed using BUSCO v5.2.2 (Manni et al., 2021) against the eudicots_odb10 dataset. QV, *k*-mer completeness and *k*-mer copy number plots were estimated and generated using merqury v1.3 (Rhie et al., 2020). The 12 largest scaffolds corresponding to the 12 chromosomes of *Silene acaulis* within hap1 (eth_SilAcau_F_GE_1.fa) and hap2 (eth_SilAcau_F_GE_2.fa) assemblies were aligned to one another using minimap2 (Li, 2018) and are paired as scaffold groups (SG) based on the best alignments.

Reference genome annotation

De novo repeat families were discovered for each haplotype assembly with RepeatModeler2 (Flynn et al., 2020) and the custom library used to soft mask repeat sequences with RepeatMasker (Smit et al., 2013). RNA was extracted from snap frozen leaf, bud and capsule tissues of a female individual of closely related *Silene acaulis* subsp. *exscapa*. The RNA libraries were produced based on the Illumina TruSeq Stranded mRNA kit and 150bp paired-end reads were sequenced. A total of 216,988,554 RNA-seq read pairs, the Viridiplantae partition of OrthoDB v11 (Kuznetsov et al., 2023) protein sequences and the soft masked haplotype assemblies were used as input for protein coding gene model prediction using the BRAKER3 pipeline (Gabriel et al., 2024). The resulting gene models were further filtered to remove malformed models with gffread (Pertea & Pertea, 2020). The annotation was assessed in several ways. Basic descriptive statistics of gene models including the ratio of mono- to

multi-exonic gene models (mono:multi ratio) were calculated in R v4.1.3. A mono:multi ratio around 0.2 is considered optimal (Vuruputoor et al., 2023). Amino acid sequences based on predicted gene models were assessed for completeness using BUSCO v5.2.2 (Mosè Manni et al., 2021) in protein mode and searched against the eggNOG v5 (Huerta-Cepas et al., 2018) database for sequence similarity hits and to assign putative functional descriptions using eggNOG-mapper v2 (Cantalapiedra et al., 2021).

Supplementary Figures

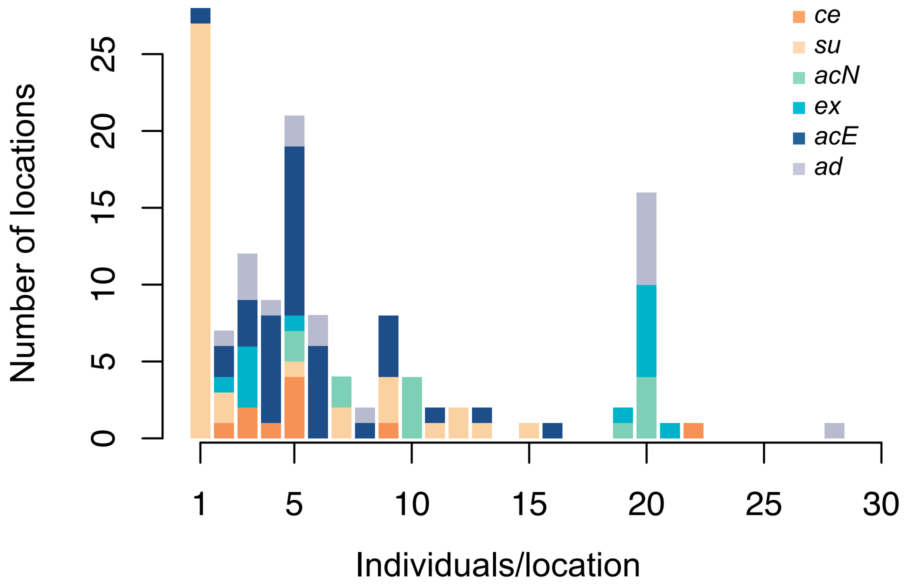

Figure S1: Histogram showing the distribution of the 955 individuals of the *Silene acaulis* species complex included in the genetic analysis across 132 sampled locations, based on the number of individuals per location. The colors represent the genetic groups to which the individuals belong, with group assignments indicated in the legend. The x-axis denotes the number of individuals sampled per location, and the y-axis shows the corresponding number of locations.

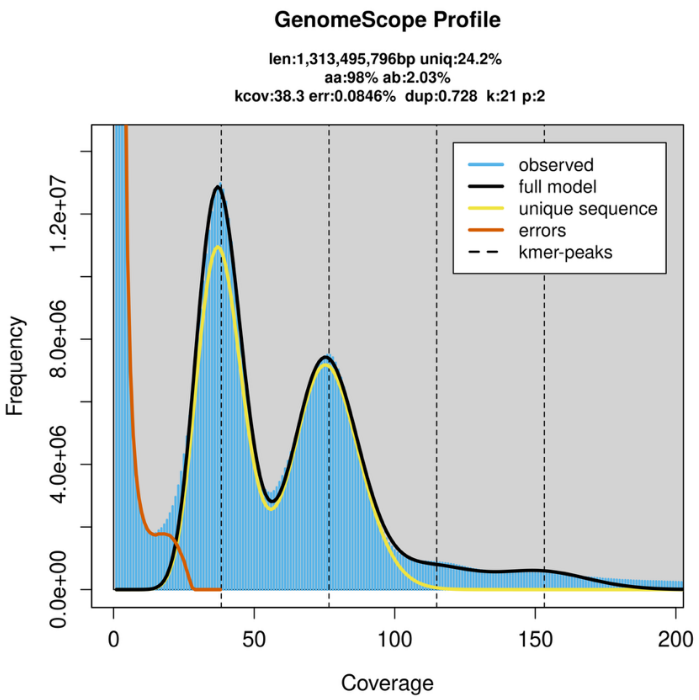

Figure S2: Genome size and heterozygosity estimation for *Silene acaulis*. The GenomeScope profile shows *k*-mer-based estimates of genome size (len), heterozygosity (ab) and repeat content (100-uniq) from the frequency of *k*-mers ($k = 21$) within HiFi reads.

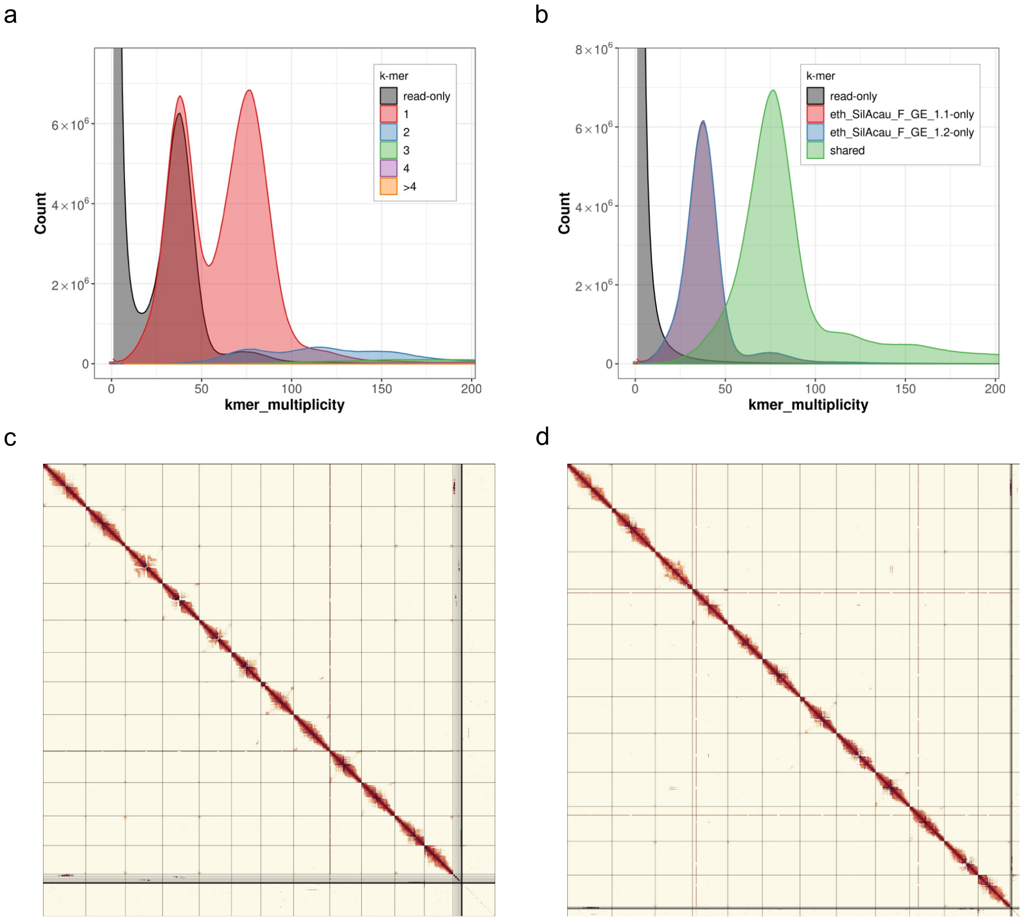

Figure S3: Reference genome assembly evaluation. **a**) The assembly *k*-mer ($k = 21$) copy number spectrum shows few errors and low level of duplications within eth_SilAcau_F_GE_1.fa, the reference for this study. **b**) The shared *k*-mer copy number spectrum shows even distribution of heterozygous *k*-mers between the two haplotype assemblies uploaded. Omni-c contact maps show strong contact frequency along the diagonal within the 12 scaffold groups (leftmost squares) of **c**) eth_SilAcau_F_GE_1 and **d**) eth_SilAcau_F_GE_2 corresponding to chromosomes. Scaffold groups are ordered in ascending order from left to right; SG_1 to SG_12. Unplaced scaffolds are also depicted.

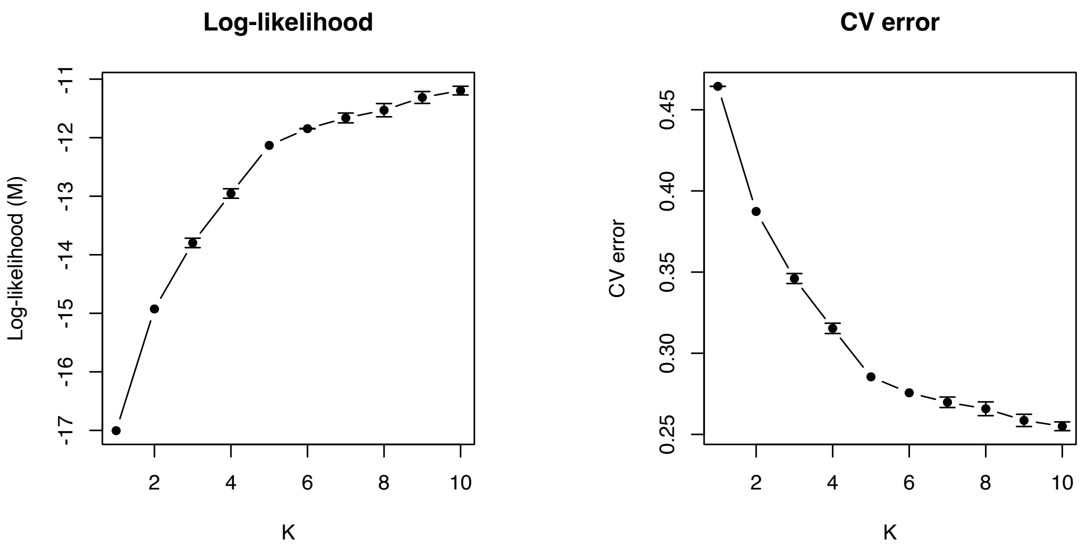

Figure S4: Log-likelihood values (left) and cross-validation (CV) errors (right) resulting from ADMIXTURE analysis for K = 1-10 ancestral populations with 955 individuals and 28,666 SNPs. Error bars indicate the standard deviation based on 10 repetitions.

Major modes for the uploaded data:

K=1

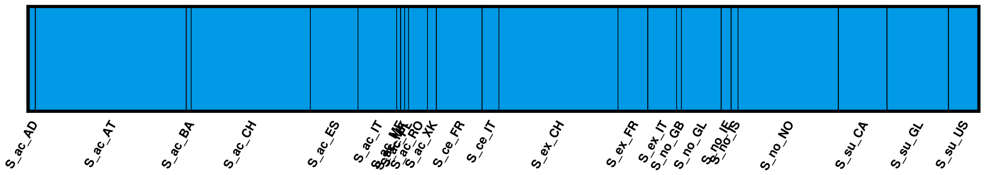

K=2

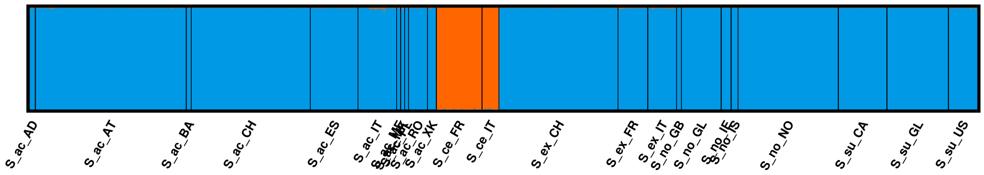

K=3

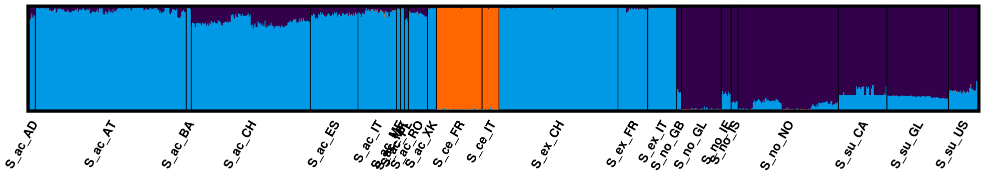

K=4

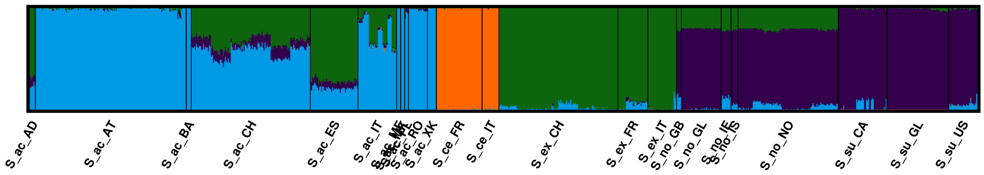

K=5

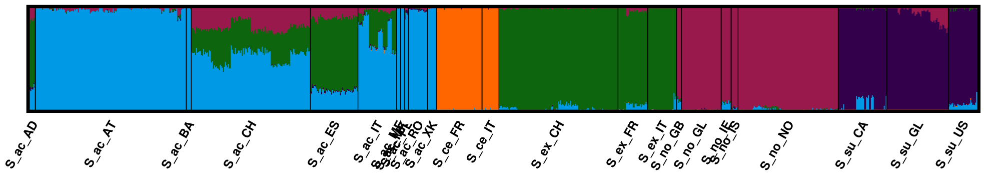

K=6

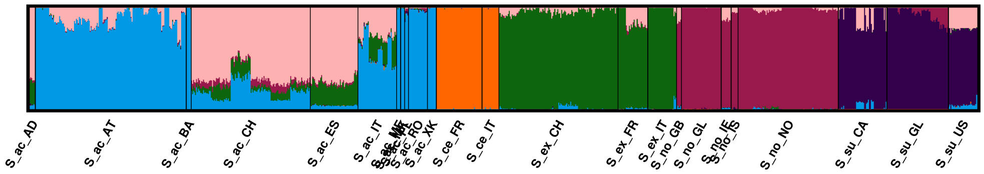

K=7

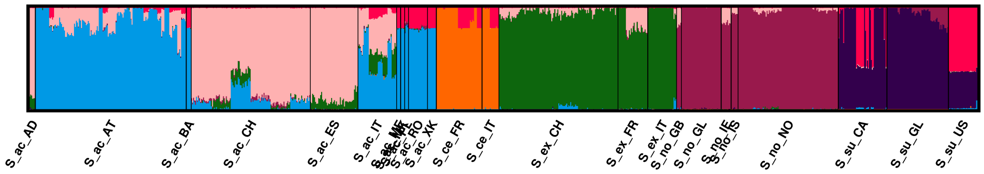

K=8

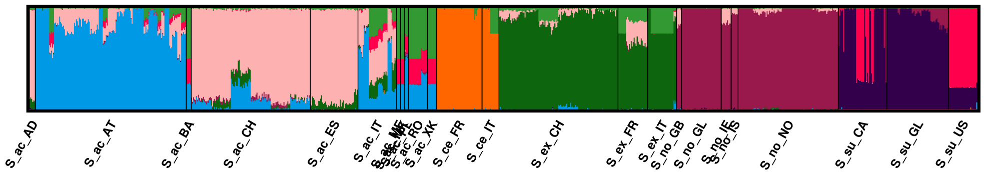

K=9

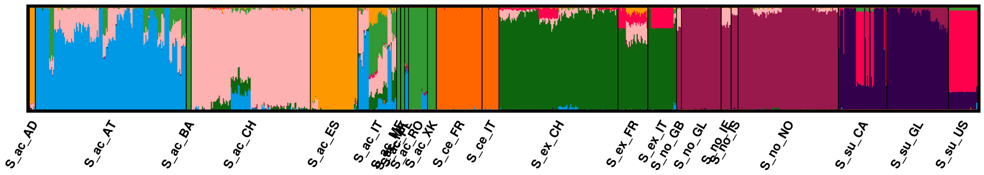

K=10

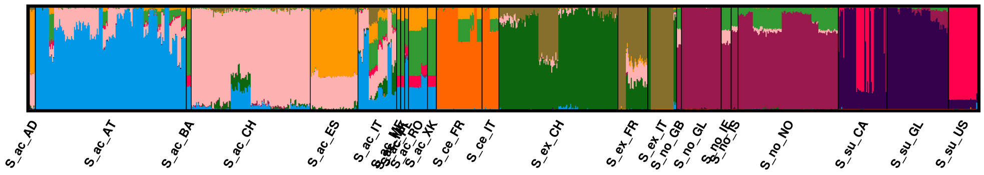

Minor modes for the uploaded data:

K=3   MinorCluster1

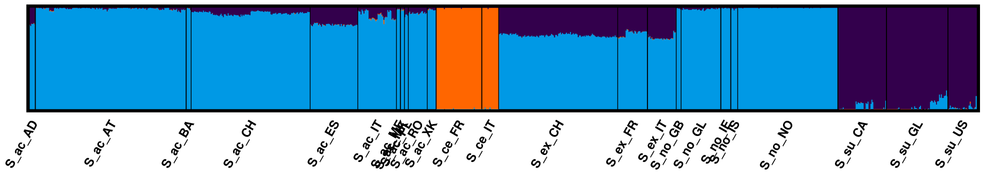

K=4   MinorCluster1

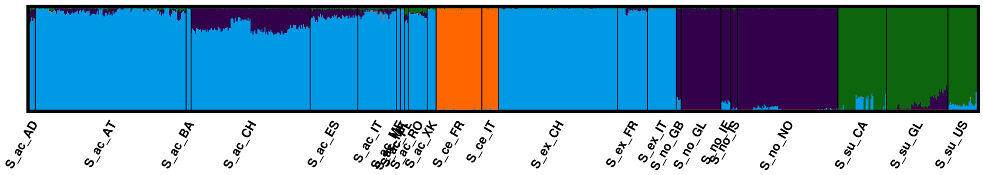

K=4   MinorCluster2

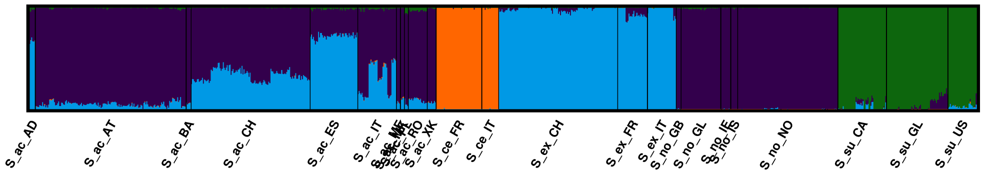

K=7   MinorCluster1

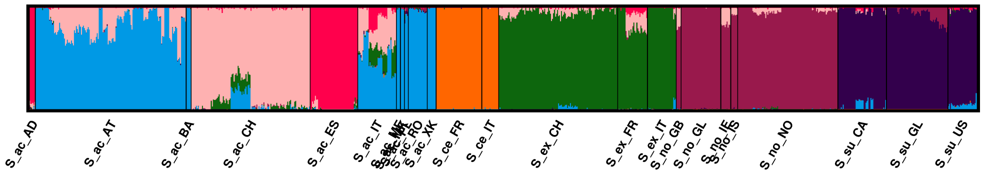

K=7   MinorCluster2

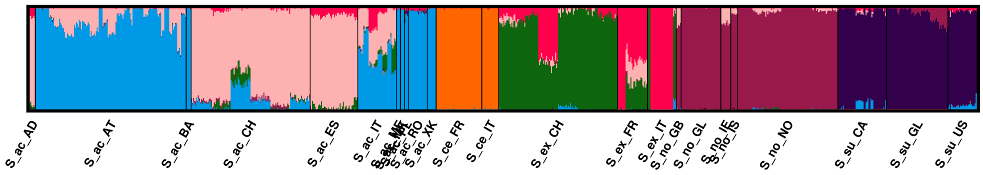

K=8 MinorCluster1

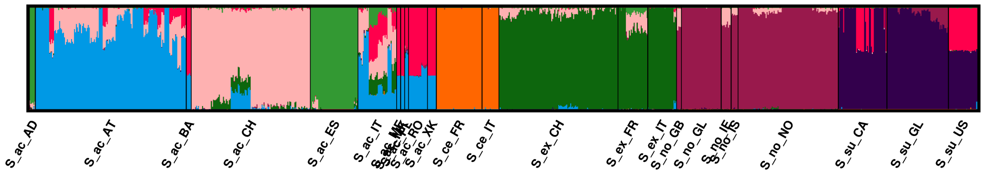

K=8 MinorCluster2

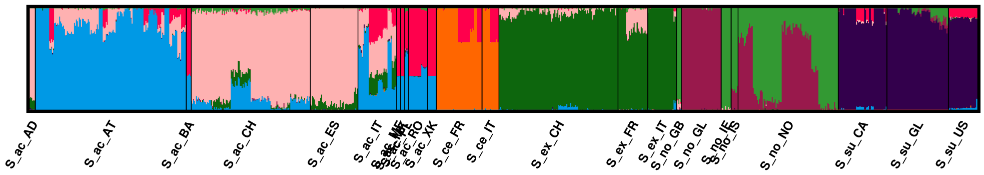

K=9 MinorCluster1

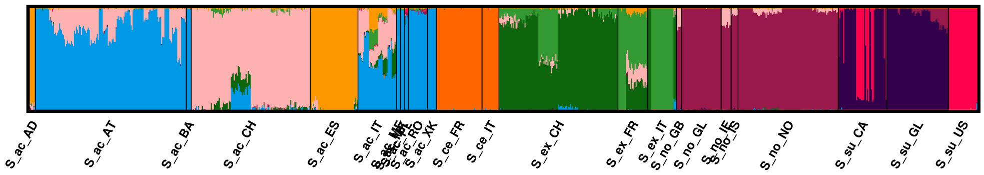

K=9 MinorCluster2

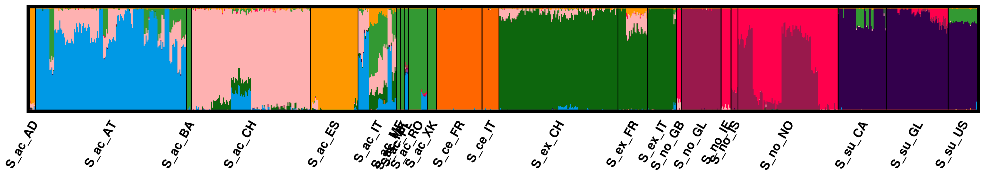

K=9 MinorCluster3

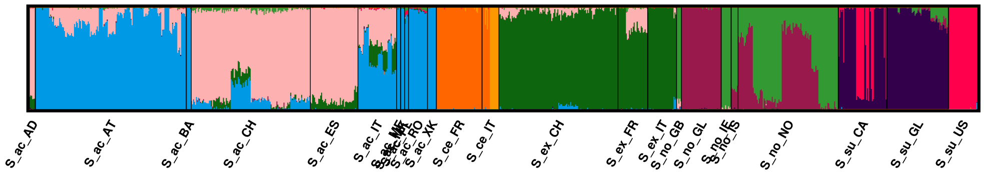

K=10 MinorCluster1

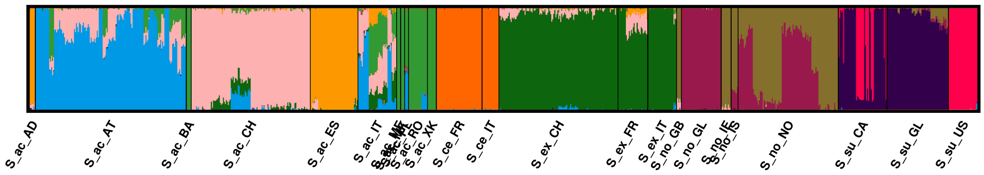

Division of runs by mode:

K=1	10/10
K=2	10/10
K=3	7/10, 3/10
K=4	5/10, 3/10, 2/10
K=5	10/10
K=6	10/10
K=7	5/10, 3/10, 2/10
K=8	4/10, 3/10, 3/10
K=9	5/10, 2/10, 2/10, 1/10
K=10	9/10, 1/10

Figure S5: CLUMPAK output for admixture runs for the ADMIXTURE analysis for K = 2-10 ancestral populations using 955 individuals of the *Silene acaulis* species complex and 28,666 SNPs. Shown are the minor and major modes of the runs and the divisions of minor and major modes. Samples are grouped according to their country of origin.

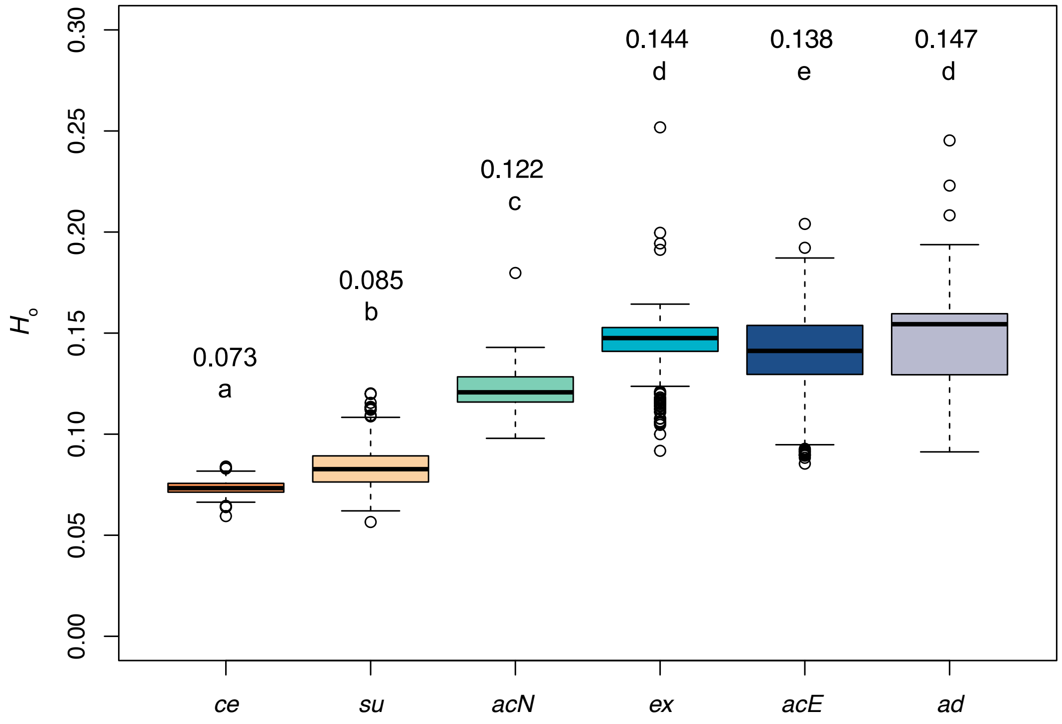

Figure S6: Boxplot illustrating the distribution of observed heterozygosity (H_O) values across genetic groups. Boxes represent the interquartile range with median values shown as horizontal lines. Mean values are displayed above each box, and letters indicate statistically significant differences between groups based on ANOVA.

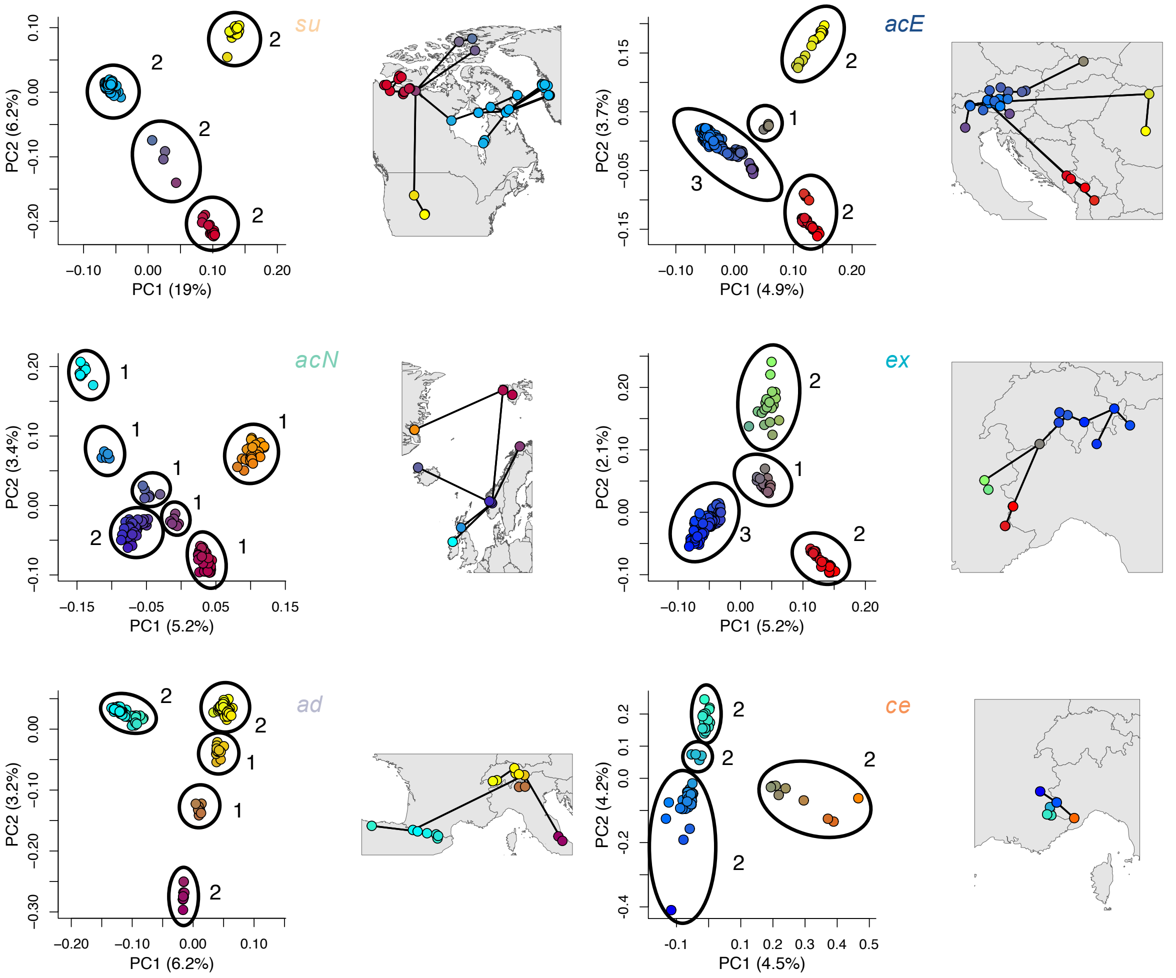

Figure S7: Representative selection of samples within the different evolutionary lineages of the *Silene acaulis* species complex for the phylogenetic analyses. For each genetic group, a PCA and map with sampling locations coloured according to average PCA values and connected based on a minimum spanning tree is shown. Black circles indicate the genetic clusters from which we selected a certain number of samples. We aimed to select an equal number of individuals per PCA cluster, totalling to eight samples. Clusters represented by less populations were less weighted, if the total number of clusters was not divisible by eight. Samples were selected based on highest coverage and, if possible, different populations were considered.

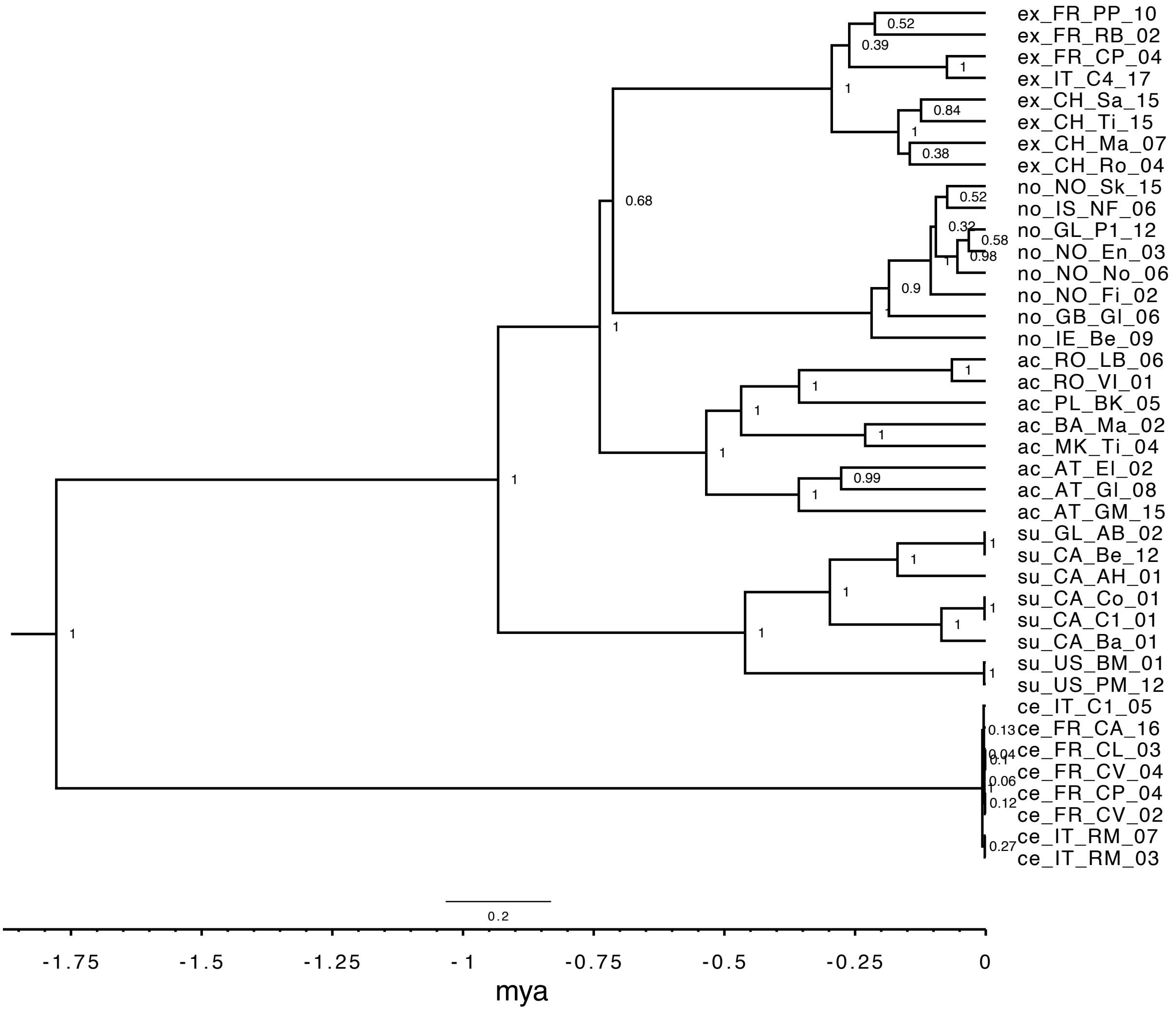

Figure S8: Phylogenetic tree of the *Silene acaulis* species complex inferred using SNAPPER in BEAST, based on 5,176 LD-pruned SNPs from 40 individuals. Posterior node support values are indicated with numbers; the scale is given in units of million years ago (mya).

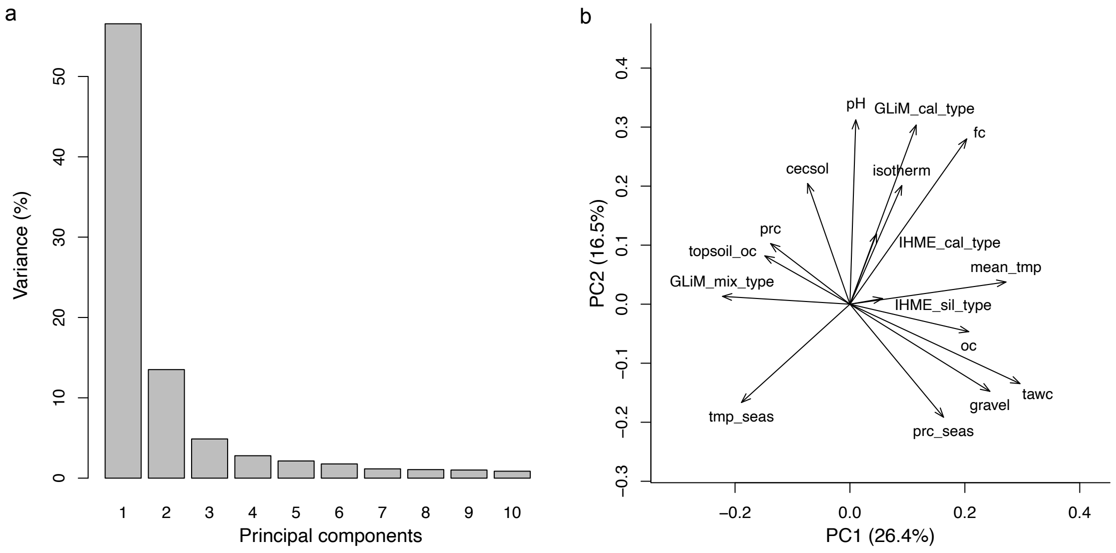

Figure S9: Principal component analyses of genetic and environmental variation. **a**) Proportion of variance explained by the first ten principal components derived from a PCA of allele frequencies across 41 populations in the European Alps. Bars indicate the relative contribution of each component to the total genetic variation. **b**) PCA biplot based on 16 uncorrelated environmental variables. Arrows indicate the relative strength and direction of each variable's contribution to the principal components (variable abbreviations are provided in Table S7).

References

Cantalapiedra, C. P., Hernández-Plaza, A., Letunic, I., Bork, P., & Huerta-Cepas, J. (2021). eggNOG-mapper v2: functional annotation, orthology assignments, and domain prediction at the metagenomic scale. *bioRxiv*, 2021.2006.2003.446934. doi:10.1101/2021.06.03.446934

Cheng, H., Jarvis, E. D., Fedrigo, O., Koepfli, K.-P., Urban, L., Gemmell, N. J., & Li, H. (2022). Haplotype-resolved assembly of diploid genomes without parental data. *Nature Biotechnology, 40*(9), 1332-1335. doi:10.1038/s41587-022-01261-x

Flynn, J. M., Hubley, R., Goubert, C., Rosen, J., Clark, A. G., Feschotte, C., & Smit, A. F. (2020). RepeatModeler2 for automated genomic discovery of transposable element families. *Proceedings of the National Academy of Sciences, 117*(17), 9451-9457. doi:10.1073/pnas.1921046117

Gabriel, L., Brůna, T., Hoff, K. J., Ebel, M., Lomsadze, A., Borodovsky, M., & Stanke, M. (2024). BRAKER3: Fully automated genome annotation using RNA-seq and protein evidence with GeneMark-ETP, AUGUSTUS and TSEBRA. *bioRxiv*, 2023.2006.2010.544449. doi:10.1101/2023.06.10.544449

Huerta-Cepas, J., Szklarczyk, D., Heller, D., Hernández-Plaza, A., Forslund, S., Cook, H., . . . Bork, P. (2018). eggNOG 5.0: a hierarchical, functionally and phylogenetically annotated orthology resource based on 5090 organisms and 2502 viruses. *Nucleic Acids Research, 47*. doi:10.1093/nar/gky1085

Kuznetsov, D., Tegenfeldt, F., Manni, M., Seppey, M., Berkeley, M., Kriventseva, Evgenia V., & Zdobnov, E. M. (2023). OrthoDB v11: annotation of orthologs in the widest sampling of organismal diversity. *Nucleic Acids Research, 51*(1), 445-451. doi:10.1093/nar/gkac998

Li, H. (2018). Minimap2: pairwise alignment for nucleotide sequences. *Bioinformatics, 34*(18), 3094-3100. doi:10.1093/bioinformatics/bty191

Manni, M., Berkeley, M. R., Seppey, M., Simão, F. A., & Zdobnov, E. M. (2021). BUSCO update: Novel and streamlined workflows along with broader and deeper phylogenetic coverage for scoring of eukaryotic, prokaryotic, and viral Genomes. *Molecular Biology and Evolution, 38*(10), 4647-4654. doi:10.1093/molbev/msab199

Pertea, G., & Pertea, M. (2020). GFF Utilities: GffRead and GffCompare. *F1000Res, 9*. doi:10.12688/f1000research.23297.2

Ranallo-Benavidez, T. R., Jaron, K. S., & Schatz, M. C. (2020). GenomeScope 2.0 and Smudgeplot for reference-free profiling of polyploid genomes. *Nature Communications, 11*(1), 1432. doi:10.1038/s41467-020-14998-3

Rhie, A., Walenz, B. P., Koren, S., & Phillippy, A. M. (2020). Merqury: Reference-free quality, completeness, and phasing assessment for genome assemblies. *Genome Biology, 21*(1), 245. doi:10.1186/s13059-020-02134-9

Smit, A., Hubley, R., & Green, P. (2013). RepeatMasker Open-4.0.

Vuruputoor, V. S., Monyak, D., Fetter, K. C., Webster, C., Bhattarai, A., Shrestha, B., . . . Wegrzyn, J. L. (2023). Welcome to the big leaves: best practices for improving genome annotation in non-model plant genomes. *Applications in Plant Sciences, 11*(4), e11533. doi:10.1002/aps3.11533

Zhou, C., McCarthy, S. A., & Durbin, R. (2023). YaHS: Yet another Hi-C scaffolding tool. *Bioinformatics, 39*(1), btac808. doi:10.1093/bioinformatics/btac808
